# Explainability and Trust in Deep Learning for Cancer Imaging: Systematic Barriers, Clinical Misalignment, and a Translational Roadmap

**DOI:** 10.3390/cancers18091361

**Published:** 2026-04-24

**Authors:** Surekha Borra, Nilanjan Dey, Simon Fong, R. Simon Sherratt, Fuqian Shi

**Affiliations:** 1 Department of CSE (ICB), K.S. Institute of Technology, Bangalore 560109, India; surekhaborra@ksit.edu.in; 2 Department of CSE, Techno International New Town, Kolkata 700156, India; nilanjan.dey@tint.edu.in; 3 Department of Computer and Information Science, Faculty of Science and Technology, University of Macau, Taipa, Macau 999078, China; ccfong@umac.mo; 4 Department of Biomedical Engineering, University of Reading, Reading RG6 6AY, UK; r.s.sherratt@reading.ac.uk; 5 Laura and Isaac Perlmutter Cancer Center, NYU Langone Health, New York University, New York, NY 10016, USA

**Keywords:** deep learning, cancer imaging, explainable artificial intelligence, clinical trust, uncertainty calibration, human–AI collaboration, regulatory governance, algorithmic bias

## Abstract

Deep learning (DL) has transformed cancer imaging by enabling automated tumour detection, classification, and risk prediction. Despite impressive diagnostic performance, limited explainability and poor uncertainty calibration continue to restrict clinical integration. This review is guided by five research questions that examine the challenges, impact, and translational implications of explainable artificial intelligence (XAI) in oncology imaging. We identify key barriers to trust, including dataset bias, shortcut learning, opacity of convolutional neural networks, and workflow misalignment. Evidence suggests that explainable models can increase clinician confidence, reduce false positives, and improve collaborative decision-making when explanations are faithful, semantically meaningful, and uncertainty aware. We evaluate architectural strategies that embed interpretability such as concept-bottleneck models, prototype-based learning, and attention regularization along with post hoc techniques. Beyond performance metrics, we examine how interpretable AI aligns with clinical reasoning processes and analyse regulatory, ethical, and medico-legal considerations influencing deployment. The findings indicate that explainability alone is insufficient, durable trust requires epistemic alignment, prospective validation, lifecycle governance, and equity-focused evaluation. By reframing explainability as a structural design principle rather than a supplementary feature, this review outlines a pathway toward accountable and clinically dependable AI systems in oncology.

## Simple Summary

Deep learning has improved cancer imaging, but many models remain “black boxes,” limiting clinical trust. Issues such as hidden bias and poor performance across hospitals further hinder real-world use. This study examines key barriers to trust and reviews methods to improve transparency, interpretability, and reliability. It emphasizes aligning artificial intelligence with clinical reasoning and communicating uncertainty. By focusing on robustness rather than accuracy alone, this work supports the development of trustworthy tools for cancer diagnosis and care.

## 1. Introduction

Cancer remains a leading cause of global mortality, with the number of new cases estimated to increase to 35 million annually by 2050 [1]. In contemporary oncology, diagnostic precision determines therapeutic trajectories, survival, and long-term quality of life. The field has evolved into a multimodal enterprise that integrates structural assessment via computed tomography (CT), soft-tissue characterisation through magnetic resonance imaging (MRI), metabolic profiling with positron emission tomography (PET), and cellular grading through digital pathology [2]. Clinical efficacy depends on detecting subtle features such as microcalcifications on mammography or irregular lesion boundaries on ultrasound that signal early disease [3]. However, the growing volume and complexity of multiparametric imaging data impose a substantial cognitive burden on radiologists, contributing to diagnostic variability and burnout [4,5].

Cancer imaging analytics have transitioned from traditional machine learning (ML), which relies on handcrafted features, to deep learning (DL). Convolutional neural networks (CNNs) autonomously learn hierarchical representations and identify complex patterns that are often imperceptible to human observers. Prospective studies report that the performance of Artificial Intelligence (AI) is comparable to, and sometimes exceeds, that of board-certified radiologists in terms of mammographic screening and lung nodule classification [6]. Architectures such as ResNet50 and DenseNet121 achieve accuracies above 94% in brain tumour classification [7], whereas vision transformers (ViTs) address the spatial limitations of convolutional filters by modelling global contextual dependencies [8]. Models may rely on spurious correlations rather than meaningful pathological features, raising safety concerns in high-stakes oncology settings. Further, despite their high performance, the “black box” nature of models may remain as a major barrier to clinical adoption [9]. Today, clinical decision-making extends beyond binary classification and requires reasoning about tumour staging, aggressiveness, and therapeutic response [10]. Therefore, for AI systems to function as credible clinical partners, their outputs must be verifiable against established medical guidelines and align with domain-specific reasoning processes [11].

A critical distinction in this context lies between interpretability and explainability. Interpretability refers to models that are transparent by design, enabling clinicians to trace how inputs are transformed into outputs. Examples include linear models or decision trees incorporating biologically grounded constraints. However, such models often lack the representational capacity required for high-dimensional imaging data [12]. Explainability, by contrast, involves post hoc techniques applied to complex deep networks whose internal reasoning is not inherently transparent. Methods such as saliency maps, class activation mapping, and feature attribution approximate which regions or features influence predictions [13]. This distinction highlights a fundamental trade-off between predictive performance and transparency, necessitating auxiliary explanation mechanisms to support accountability and trust [14].

The imperative for explainability extends beyond technical transparency to clinical, ethical, and regulatory domains. Clinically, explanations allow physicians to validate predictions against established knowledge and detect reliance on artifacts or institutional biases [15]. Without such insight, models risk being “right for the wrong reason.” Ethically, opacity complicates professional accountability, as clinicians cannot justify or contest recommendations, they do not understand [16]. Limited transparency may also increase automation bias and undermine informed consent in high-stakes decisions.

Regulatory bodies on the other hand increasingly codify these expectations. The U.S. Food and Drug Administration’s Action Plan for AI/ML-based Software as a Medical Device (SaMD) and the European Union’s General Data Protection Regulation (GDPR) emphasize transparency, documentation, and human oversight for high-risk AI systems [17]. Noncompliance may hinder regulatory approval and real-world deployment.

Trust in cancer imaging AI should therefore be conceptualised not as a static software attribute but as a calibrated, relational process between clinicians and algorithmic systems. Establishing such trust requires technical robustness, meaningful explanations, alignment with clinical reasoning, and demonstrable benefits to human decision-making.

This review paper investigates the technical and sociotechnical barriers to integrating explainable artificial intelligence (XAI) into cancer imaging from 2023 to 2025. It evaluates architectural and post hoc modelling strategies to determine how they can improve clinician trust, diagnostic accuracy, and alignment with medical reasoning. The study is guided by the following research questions:

- RQ1. What are the key challenges limiting explainability and trust in deep learning–based cancer imaging analysis?
- RQ2. Do XAI methods measurably increase clinician trust in deep learning models for malignant tumour detection?
- RQ3. Which deep learning techniques and architectural modifications improve the interpretability of CNN-based cancer image classifiers?
- RQ4. How do interpretable AI methods align with clinical reasoning and decision-making in oncology imaging?
- RQ5. To what extent do XAI approaches improve diagnostic performance and clinician confidence compared with black-box models?

This review distinguishes itself from existing surveys on XAI in oncology by shifting the focus from purely algorithmic performance to a multidimensional, lifecycle-based analysis of trust. While previous works often treat explainability as a supplementary feature, this study reframes it as a structural design principle essential for clinical integration. The unique contributions of this work include:

- Temporal and Sociotechnical Scope: We specifically synthesise developments from 2023 to 2025, moving beyond standard performance metrics to evaluate robustness, fairness, and human-centred trust.
- The “Trust-Critical Lifecycle” Framework: Unlike traditional technical overviews, this review conceptualises trust as a dynamic, system-level property shaped by every stage from data acquisition and annotation to post-deployment monitoring.
- Clinical–Epistemic Alignment: A core novelty is our investigation into whether algorithmic reasoning structurally matches the hierarchical and contextual reasoning used by clinicians, rather than just providing visual heatmaps.
- Translational Roadmap: We provide a strategic pathway that integrates technical architectures (intrinsic vs. post hoc) with regulatory evolution (e.g., FDA PCCP and EU AI Act), ethical accountability, and medico-legal liability.

To ensure a comprehensive and systematic synthesis of the field, the following methodology was employed to identify and select the literature presented in this review.

Search Strategy and Information Sources: The study identifies and synthesises technical and sociotechnical developments published between 2023 and 2025. A systematic search was conducted across primary scientific databases, including PubMed, IEEE Xplore, Google Scholar, and Scopus, to address five core research questions (RQ1–RQ5) regarding explainability and trust in oncology AI.

Search Criteria and Keywords: The search utilised a combination of Boolean operators and the following primary keywords: “Deep learning,” “cancer imaging,” “explainable artificial intelligence (XAI),” “clinical trust,” “uncertainty calibration,” “human–AI collaboration,” “regulatory governance,” and “algorithmic bias”.

Inclusion and Exclusion Criteria: Studies were selected based on their relevance to the diagnostic and therapeutic continuum of cancer care.

Inclusion Criteria:

- Research focusing on deep learning architectures (CNNs, Vision Transformers, GNNs) applied to oncology imaging.
- Studies addressing core computational tasks: tumour detection, segmentation, classification, and prognostic modelling.
- Papers evaluating interpretability methods and their impact on clinical reasoning or clinician confidence.
- Articles discussing regulatory frameworks (FDA, GDPR) and ethical considerations in AI deployment.

Exclusion Criteria:

- General AI studies without specific application to cancer-specific imaging.
- Papers focusing exclusively on predictive accuracy without addressing explainability, robustness, or trust.
- Research published prior to 2023, unless providing a foundational technical lineage.

Data Extraction and Synthesis: Selected literature was categorised into a structured taxonomy to bridge the gap between technical performance and clinical utility.

The remainder of this review is structured to progressively bridge technical performance with clinical trust. Section 2 establishes foundational concepts, outlining imaging modalities, computational paradigms, and core oncological tasks that define the operational landscape of AI in cancer imaging. Section 3 examines system-level barriers that undermine trust, including data-centric vulnerabilities, robustness limitations, fairness concerns, and the failure modes of current explainability techniques. Section 4 synthesizes contemporary explainable and interpretable modelling strategies, analysing their methodological foundations and alignment with clinical reasoning. Section 5 evaluates human–AI trust calibration, exploring uncertainty quantification and validation beyond standard accuracy. It further synthesises results from prospective trials (e.g., MASAI, NELSON) and categorises systemic barriers such as technical fragility, cognitive load exploitation, and clinical–epistemic misalignment Section 6 establishes a translational roadmap by integrating regulatory evolutions (FDA PCCP, EU AI Act) and ethical-legal considerations. Finally, it provides an integrative synthesis of RQ1–RQ5 and proposes a progressive model for trust integration before concluding in Section 7.

## 2. Foundations and Computational Tasks in Cancer Imaging

This section provides a structured overview of how deep learning has transformed cancer imaging, establishing the conceptual and methodological foundations that underpin current research and clinical applications. It introduces the key data modalities, architectural paradigms, and computational tasks that define modern oncological imaging pipelines, setting the stage for a detailed examination of how these components interact to support accurate, interpretable, and clinically actionable decision-making.

### 2.1. Imaging Modalities and Data Ecosystems

Deep learning architectures in oncology are being increasingly shaped by a triangulated ecosystem of data sources: radiological imaging, digital pathology, and longitudinal clinical trajectories [18]. Model performance and reliability are governed by the spatial resolution, biological specificity, and temporal structure of these modalities, each of which introduces distinct challenges related to acquisition variability, annotation constraints, noise, and interpretability. Understanding modality-specific properties is therefore essential, as they directly influence downstream robustness, generalizability, and explainability. This section outlines the principal imaging modalities in oncological AI and the structural constraints that shape trustworthy deployment.

#### 2.1.1. Radiological Interpretability

Radiological modalities including CT, MRI, PET form the anatomical and metabolic backbone of non-invasive cancer staging and treatment planning.

CT is widely utilized because of its high-resolution anatomical detail and rapid acquisition. In cancer diagnostics, convolutional neural networks have shifted analysis from handcrafted radiomic feature extraction to automated representation learning, enabling the detection of subtle architectural distortions that are often imperceptible to human observers. However, CT-based models are highly sensitive to acquisition parameters, including the radiation dose, reconstruction kernel, and contrast timing. Such variability introduces interscan heterogeneity that can significantly undermine cross-institutional generalizability. These effects illustrate how modality-specific physics directly constrains model robustness and contributes to domain shift.

MRI provides superior soft-tissue contrast and is central to neuro-oncology and prostate cancer imaging. Deep learning systems including federated learning configurations integrated with architectures such as GoogLeNet have demonstrated classification accuracies approaching 94% for brain tumour analysis [19]. Nevertheless, clinical reliability remains limited by scanner-dependent variability. Differences in vendor hardware, pulse sequences, and coil configurations can significantly shift feature distributions, leading to measurable performance degradation. For instance, a multicentre study using the ProstateNet dataset (comprising 5478 biparametric MRI studies from 13 European centres) demonstrated that variations between Siemens, Philips, and GE manufacturers, alongside the use of endorectal coils (ERC), resulted in an average AUC reduction of 0.05. This occurred when models trained on data from one specific manufacturer or protocol were evaluated on external data from another, highlighting that scanner-specific hurdles can impede the generalisability of models designed to classify prostate cancer aggressiveness [20]. These findings underscore the need for harmonization and domain adaptation to maintain reproducible performance across institutions.

PET contributes complementary metabolic and molecular information through radiotracer uptake patterns. To improve image quality under accelerated acquisition protocols, generative adversarial networks (GANs), including modified pix2pixHD variants, have been employed to enhance ultrafast PSMA-PET images, yielding reported improvements of 17.9% in region detection rates [21]. However, while generative enhancement can improve visual fidelity and apparent lesion conspicuity, it cannot reconstruct metabolic information that was not captured during acquisition. Consequently, small lesions with low radiotracer uptake remain challenging, highlighting the information-theoretic limits of post-acquisition reconstruction and reinforcing the need for cautious clinical validation.

Collectively, radiological modalities demonstrate how acquisition physics, vendor heterogeneity, and signal constraints directly affect model generalizability, reliability, and interpretability.

#### 2.1.2. Digital Pathology Efficiency

While radiological imaging is shaped primarily by acquisition physics and scanner variability, digital pathology presents a distinct computational landscape defined by extreme scale and annotation sparsity. The digitization of histopathology through whole-slide imaging (WSI) has enabled quantitative, gigapixel-scale computational analysis. However, these images frequently exceed billions of pixels, making direct end-to-end processing computationally impractical [22]. As a result, most pipelines rely on structured data reduction strategies.

Multiple instance learning (MIL) has emerged as a dominant framework, aggregating patch-level representations to generate slide-level predictions without requiring exhaustive pixelwise annotation [23]. To mitigate the dilution of diagnostically relevant features by extensive noninformative tissue, approaches such as evolutionary patch selection (EvoPS) apply multiobjective optimization to retain informative regions selectively. This strategy has been shown to reduce data requirements by more than 90% while preserving classification performance [24]. Such approaches illustrate how intelligent sampling can enhance both efficiency and signal fidelity.

Architectural evolution is also reshaping computational pathology. Transformer-based models, including hierarchical variants such as HIPT, capture multiscale representations from cellular morphology to global tissue architecture. State-space approaches, such as Vision Mamba, further improve scalability by avoiding the quadratic complexity of standard self-attention mechanisms, enabling efficient whole-slide analysis [25].

Despite these advances, digital pathology remains constrained by annotation bottlenecks, interobserver variability, and stain heterogeneity factors that directly affect label integrity and downstream model trustworthiness. These structural challenges are closely linked to the data-centric barriers discussed in subsequent sections.

#### 2.1.3. Multimodal and Longitudinal Synthesis

Precision oncology increasingly depends on the integration of heterogeneous data sources, including imaging, genomics, and longitudinal clinical records, to construct comprehensive representations of tumour biology. Multimodal fusion frameworks such as SMuRF, which are based on Swin Transformer architectures, employ cross-modality attention to align macroscale radiological patterns with microscopic pathological features. In oropharyngeal squamous cell carcinoma, such approaches have achieved a concordance index (C-index) of 0.81, outperforming unimodal baselines [26].

Heterogeneous data structures, variable sampling frequencies, and frequent missing data in real-world clinical datasets complicate training and validation during multimodal integration. Moreover, multimodal models are often developed on limited paired datasets, raising concerns regarding overfitting and statistical fragility. Robust evaluation across heterogeneous cohorts remains essential for reliable clinical translation.

Beyond cross-sectional fusion, modelling longitudinal dynamics provides insight into tumour evolution and treatment response. “Delta-radiomics,” which captures temporal variation in imaging features, has demonstrated value in predicting immunotherapy outcomes. Methods such as LILAC (Learning-based Inference of Longitudinal ImAge Changes) employ shared CNN or Siamese architectures to localize and quantify patient-specific temporal changes [27]. By learning temporal ordering and associated clinical score trajectories, these approaches reveal disease progression patterns that may be obscured in static analyses [28].

Temporal modelling may also support internal consistency checks across time points, offering an additional dimension for assessing prediction stability. Nevertheless, longitudinal datasets are often incomplete and irregularly sampled, presenting challenges for robust training and clinical validation.

Collectively, these imaging ecosystems define the data substrate of oncological AI and the constraints within which computational models must operate. Differences in modality physics, resolution, annotation granularity, and clinical workflow shape how information is encoded and interpreted. Accordingly, the evolution of deep learning paradigms in cancer imaging cannot be understood independently of these structural conditions. The following section examines how architectural innovations have emerged in response to these demands.

### 2.2. Deep Learning Paradigms in the Clinical Context

The evolution of deep learning in oncological imaging mirrors the broader transformation of cancer imaging ecosystems described in Section 2.1. As imaging modalities generate increasingly high-dimensional, multimodal, and longitudinal data streams, computational frameworks have shifted from isolated pixel-level pattern recognition toward architectures capable of modelling structural, contextual, and biological relationships. Unlike traditional computer-aided diagnosis (CAD), which relies on handcrafted features constrained by predefined assumptions, modern DL systems learn hierarchical representations directly from data. These representations approximate layered visual abstraction, enabling the detection of subtle anatomical and pathological patterns that may elude manual specification.

However, architectural sophistication alone does not guarantee clinical alignment. As DL systems become embedded within diagnostic and prognostic workflows, ensuring that mathematical optimization corresponds to clinically meaningful reasoning becomes a central challenge. Model design must therefore be evaluated not only in terms of predictive performance but also with respect to spatial reasoning capacity, data efficiency, biological plausibility, and interpretability.

Within this landscape, four architectural paradigms—convolutional, transformer-based, representation learning, and graph-based approaches, have emerged as dominant frameworks. Each responds to distinct computational and clinical constraints inherent in oncological imaging.

#### 2.2.1. Convolutional Architectures and Local Feature Hierarchies

Convolutional neural networks retain the foundational architecture in medical image analysis because of their structural inductive bias toward translational invariance. By applying shared convolutional kernels across spatial locations, CNNs construct hierarchical feature maps that progressively abstract raw pixel intensities into increasingly complex representations. Coupled with nonlinear activations such as rectified linear units (ReLU), this architecture enables discrimination between benign and malignant tissue based on learned morphological patterns.

Ensemble configurations incorporating architectures such as DenseNet, Xception, and VGG16 have demonstrated improved robustness and reduced interobserver variability. However, CNNs are intrinsically locality driven. Although deeper layers increase the effective receptive fields, convolution fundamentally prioritizes the proximal spatial structure. Consequently, long-range anatomical dependencies such as spatial relationships between primary tumours and distant metastatic sites may be insufficiently encoded. In complex oncological settings where global structural awareness informs diagnosis or staging, this architectural constraint can limit comprehensive context modelling [29].

#### 2.2.2. Transformer-Based Models and Global Contextual Reasoning

To address locality constraints, vision transformers (ViTs) replace convolution with self-attention mechanisms that evaluate relationships among all image patches simultaneously. Through interactions between query, key, and value vectors, self-attention dynamically weights spatial regions based on global relevance, enabling modelling of long-range dependencies critical for integrative diagnostic reasoning [30].

Clinically, transformer-based models have demonstrated utility in tasks requiring multimodal synthesis. In oropharyngeal cancer, for example, transformer frameworks integrate imaging features with structured variables such as Human Papillomavirus (HPV) status and tumour–node–metastasis (TNM) staging, enhancing risk stratification and survival prediction [31]. However, the pure transformer architecture lacks strong spatial inductive biases and typically requires large-scale training data. In medical contexts where datasets are often limited, heterogeneous, and institution-specific these properties may constrain generalizability.

Hybrid CNN–Transformer models therefore seek architectural complementarity. Designs such as BEFUnet employ dual-branch encoders in which convolutional pathways capture fine-grained anatomical boundaries, whereas Swin Transformer branches model global semantic relationships. This integration supports a balance between local precision and contextual awareness, improving robustness across diverse imaging environments [32].

#### 2.2.3. Representation Learning Under Data Scarcity

Annotation scarcity remains a defining constraint in oncological imaging, particularly in rare cancers and early-stage disease. Representation learning frameworks address this limitation by leveraging unsupervised or self-supervised objectives. Autoencoders (AEs), for instance, reconstruct input images from compressed latent embeddings, implicitly modelling the distribution of normal anatomical structures. Regions associated with elevated reconstruction error can then be interpreted as potential pathological deviations [33].

The diagnostic fidelity of such systems depends critically on latent space design. Without sufficient constraints, autoencoders risk trivial identity mappings that preserve surface detail without encoding meaningful structures. Recent work has indicated that aligning latent entropy with the intrinsic informational complexity of healthy tissue can mitigate this risk and preserve clinically relevant anatomical patterns while maintaining sensitivity to abnormal variation. Optimal latent dimensionality is modality dependent; volumetric MRI, for example, generally necessitates higher-dimensional embeddings than planar X-ray imaging does to retain diagnostic information [34].

#### 2.2.4. Graph-Based Modelling of Tissue Architecture

In digital pathology, diagnostic inference depends not only on individual cellular morphology but also on spatial organisation within the tumour microenvironment (TME). Graph neural networks (GNNs) formalize this relational structure by representing tissue as graphs, where nodes correspond to biological entities such as cells or nuclei and edges encode spatial or functional relationships [35]. Through iterative message passing, GNNs aggregate contextual information, enabling the learning of biologically meaningful topological patterns.

Hierarchical models such as HACT-Net extend this paradigm across multiple spatial scales, linking microscopic cellular configurations to macroscopic tissue architecture. This multiscale representation mirrors the interpretive process of pathologists and enhances structural transparency relative to conventional CNNs [36]. Dual-graph frameworks such as SCUBa-Net further increase representational capacity by jointly modelling short-range cellular interactions and long-range tissue-level dependencies, facilitating comprehensive analysis across whole-slide images [37].

#### 2.2.5. Integrative Perspective

Across these paradigms, a consistent trajectory emerges from locally constrained feature extraction toward architectures capable of modelling multiscale, multimodal, and relational complexity. CNNs provide efficient morphological abstraction, transformers enable global contextual reasoning, representation learning mitigates annotation bottlenecks, and graph-based models encode biological topology. However, as architectural complexity increases, so too does the opacity of model reasoning. The tension between representational power and interpretability introduced here at the architectural level becomes central in subsequent discussions of explainability, robustness, and clinical trust.

### 2.3. Core Clinical Tasks

The application of deep learning in oncological imaging is organized around computational tasks that map directly onto the diagnostic and therapeutic continuum. From lesion detection to longitudinal prognostication, each task reflects a distinct decision stage, requiring specific forms of spatial precision, contextual reasoning, or temporal modelling. Unlike isolated benchmarks, these tasks form an interdependent computational pipeline in which upstream design choices propagate downstream, influencing reliability, interpretability, and, ultimately, clinical trust.

#### 2.3.1. Tumour Detection and Localization

Detection and localization constitute the entry point of the imaging workflow, determining whether a lesion is present and estimating its spatial position. Early and accurate identification is particularly consequential in high-risk domains such as neuro-oncology, where survival outcomes are closely tied to early intervention [38].

Architecturally, detection frameworks have evolved from two-stage proposal-based models toward unified regression paradigms such as the You Only Look Once (YOLO) family, including YOLOv11 [39]. By jointly predicting bounding boxes and class probabilities in a single forward pass, these systems enable real-time inference that is compatible with clinical hardware constraints.

However, the robustness of detection remains limited by heterogeneous tumour morphology and variability in MRI acquisition protocols. Domain-specific augmentation strategies such as Mosaic augmentation and CutMix are therefore employed to enhance generalization. Mosaic augmentation increases sensitivity across tumour sizes by synthesizing composite images, whereas CutMix promotes resilience to partial occlusion and boundary ambiguity by enforcing recognition under anatomical edge conditions. These strategies are particularly relevant for early-stage gliomas, where structural deviations may be subtle [40].

Despite technical advances, detection performance varies markedly across anatomical sites. The sensitivity frequently exceeds 90% in oesophageal and breast cancer, yet the sensitivity for central nervous system (CNS) tumours ranges from 48% to 100%, reflecting dataset composition and acquisition variability. This heterogeneity underscores the necessity of multi-institutional validation to ensure generalizability and clinical reliability [40,41,42,43,44].

#### 2.3.2. Advanced Image Segmentation for Precision Oncology

Segmentation extends detection into pixel- or voxel-level delineation, enabling fine-grained characterization of tumour subregions. Such spatial fidelity is essential for radiotherapy dose planning, surgical navigation, and longitudinal treatment monitoring.

The U-Net architecture remains a dominant benchmark because of its encoder–decoder symmetry and skip connections, which preserve high-resolution spatial information while supporting deep abstraction [45]. However, infiltrative tumour margins and long-range anatomical dependencies present challenges for purely convolutional receptive fields.

To address these limitations, attention-based and hybrid mechanisms have been introduced. Attentive U-Net (AttU-Net) incorporates attention gates that suppress irrelevant background features and amplify diagnostically salient regions, providing partial interpretability through spatial weighting [46]. Similarly, the residual attention U-shaped network (RAUNet) integrates residual learning with transformer-style components to model extended spatial dependencies which is particularly important for tumours spanning multiple anatomical compartments [30].

Clinical effectiveness is typically quantified using the Dice similarity coefficient (DSC), reflecting overlap between predicted and reference segmentations. Architectures such as ACU-Net have achieved DSCs of up to 98.77% in brain tumour segmentation, supporting reproducible delineation and reducing variability in treatment planning [45].

#### 2.3.3. Histopathological Classification and Grading

In digital pathology, classification and grading require the integration of multiscale morphological information. Diagnostic inference depends simultaneously on cellular features and broader tissue architecture, demanding architectures capable of hierarchical reasoning.

Models such as MAMILNet employ multiscale “consultation” strategies, training across magnifications ranging from 5× to 20×. This design mirrors the workflow of expert pathologists, who iteratively transition between micro and macrostructural views. Such systems have demonstrated strong performance in lung and ovarian cancer subtyping while enhancing trustworthiness through structural alignment with human interpretive processes [47].

Similarly, deep learning–based grading frameworks for squamous cell carcinoma (SCC) have achieved accuracies exceeding 98%, offering reproducible alternatives to manual grading and reducing interobserver variability [48]. Importantly, these systems operationalize morphological criteria in a standardized manner, potentially stabilizing diagnostic interpretation across institutions.

#### 2.3.4. Prognosis and Dynamic Risk Stratification

Beyond diagnostic characterization, DL increasingly supports survival prediction and therapeutic response modelling [49]. Traditional time-to-event frameworks such as the Cox proportional hazards (CPH) model provide statistical baselines but are limited in capturing nonlinear feature interactions. DeepSurv extends this framework by learning complex mappings between imaging-derived representations and hazard functions, improving predictive flexibility.

More recent approaches emphasize temporal dynamics and explainability. DySurv, built upon Conditional Variational Autoencoders (CVAEs), enables longitudinal risk estimation by incorporating evolving patient data. Unlike static risk scores, DySurv updates predictions over time and has demonstrated a 12% improvement over clinical indices such as Acute Physiology And Chronic Health Evaluation (APACHE) [50].

Multimodal fusion strategies further enhance prognostic modelling. Integrating PET/CT-derived deep features with structured clinical variables improved the AUC performance in lung cancer from 0.64 to 0.84, suggesting that DL captures latent textural and temporal patterns that are not readily discernible through conventional assessment [51]. These advances indicate a transition from static classification toward adaptive, patient-specific risk modelling.

#### 2.3.5. Auxiliary Tasks

Complementing core diagnostic and prognostic functions, DL supports auxiliary tasks that enhance data fidelity and procedural reliability. Image registration frameworks such as VoxelMorph enable real-time anatomical alignment, supporting motion tracking during radiotherapy delivery [52].

Denoising and dose-reduction strategies, including residual encoder–decoder CNNs and Wasserstein GANs (WGANs), reconstruct diagnostically meaningful images from low-dose CT acquisitions, reducing radiation exposure while preserving image quality.

Cross-modality synthesis and super resolution techniques further address incomplete or low-quality imaging. Diffusion-based models such as the CG-DDPM and CycleGAN architectures generate missing modalities or enhance spatial resolution, enabling clinically meaningful interpretation under constrained acquisition conditions [53,54].

Across these task categories, DL systems increasingly function not as isolated predictors but as interdependent components within a clinical pipeline. Detection identifies candidate regions, segmentation refines spatial boundaries, classification formalizes morphological reasoning, prognostic models extend inference into temporal domains, and auxiliary systems stabilize the imaging substrate itself. However, as task complexity increases from binary detection to dynamic risk stratification the demands on robustness, interpretability, and validation intensify. The performance gains observed under controlled experimental conditions do not automatically translate into dependable clinical integration. As models assume greater responsibility across the diagnostic and therapeutic continuum, vulnerabilities related to data integrity, distributional shifts, explainability limitations, and robustness become increasingly consequential. The transition from task optimization to real-world deployment therefore necessitates a broader examination of system-level constraints. Section 3 critically evaluates these structural barriers to trustworthy AI in cancer imaging.

Table 1 summarizes the current landscape of deep learning applications across multiple cancer types and imaging modalities. It provides a structured overview of study metadata, clinical objectives, imaging modalities, target cancer sites, and the corresponding model architectures employed. The table establishes the foundational landscape of deep learning in oncology, identifying the clinical tasks and architectural paradigms where the explainability and trust challenges addressed in Research Question 1 (RQ1) and Section 3 are most critically situated.

The data reveal that convolutional neural networks dominate classification and segmentation tasks in breast and lung imaging, while graph-based and transformer architectures are emerging for complex multimodal or volumetric analyses. This tabulated landscape illustrates both the breadth and limitations of current DL deployments, offering a foundation for assessing explainability needs and model trustworthiness in subsequent sections.

**Figure 1:**
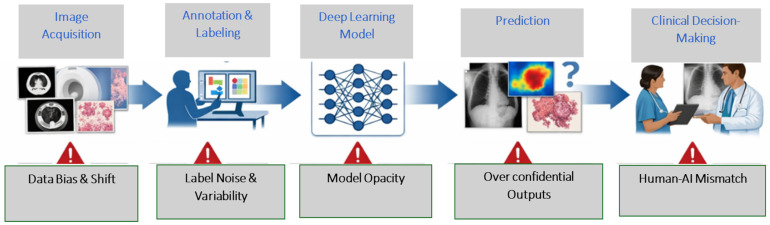
Trust-critical lifecycle of oncology imaging artificial intelligence systems.

While advances in deep learning have enabled remarkable progress in tumour detection, segmentation, classification, and prognostic modelling, the clinical translation of oncological imaging AI depends not only on its predictive accuracy but also on its sustained trustworthiness across the entire system lifecycle. Trust in medical AI is inherently multilayered, emerging from the integrity of data acquisition, annotation processes, model development, validation strategies, and clinical deployment. To contextualize these dependencies, Figure 1 presents a critical view of the cancer imaging AI pipeline, highlighting key vulnerability points across stages.

## 3. System-Level Barriers to Trustworthy AI

While the preceding section outlined the foundational components and computational capabilities of oncology imaging AI, reliable clinical translation depends on structural conditions that extend beyond algorithmic performance. In practice, trust-related challenges emerge from vulnerabilities distributed across the AI lifecycle, including data acquisition, annotation processes, model development, validation strategies, and real-world integration. Bias, limited generalizability, calibration deficiencies, interpretability constraints, and workflow misalignment may interact and compound, ultimately affecting clinical confidence and patient safety.

In high-stakes oncologic decision-making, where AI outputs may influence diagnosis, treatment planning, and prognostic assessment, isolated technical limitations can produce amplified downstream consequences. Importantly, these risks are rarely confined to a single developmental stage. Distortions introduced during data curation often remain latent during Tier 1 (internal retrospective) validation, only to surface as critical failures during Tier 2 (external multicentre) deployment or Tier 3 (prospective) clinical use. Similarly, inadequate uncertainty estimation or poorly aligned explanations may foster overreliance or inappropriate scepticism in clinical environments. Accordingly, trustworthiness should be conceptualised as a dynamic, system-level property shaped by cumulative interactions between data integrity, modelling choices, human oversight, and post deployment monitoring.

**Figure 2:**
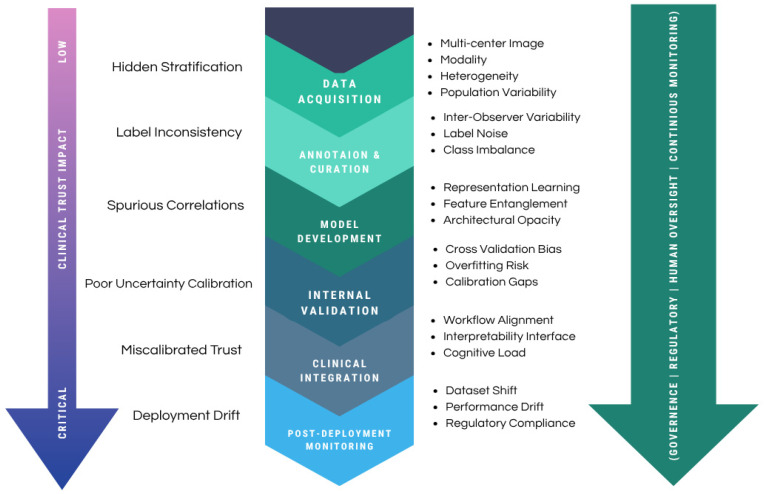
Lifecycle-integrated trust vulnerability and governance framework for oncology imaging artificial intelligence.

Figure 2 presents a lifecycle-integrated framework that maps stage-specific vulnerabilities and illustrates how unresolved weaknesses may propagate toward real-world use. The model further emphasizes the necessity of a continuous governance layer encompassing oversight, regulatory alignment, human supervision, and ongoing performance surveillance across all phases of system development and deployment. The following subsections examine these barriers in detail, beginning with interpretability and clinical trust considerations, followed by data-centric reliability challenges, robustness and fairness constraints, and limitations of current explainability approaches.

### 3.1. Interpretability, Explainability, and Clinical Trust

A foundational distinction in clinical artificial intelligence lies between interpretability and explainability, as each reflects a different design philosophy and set of clinical trade-offs. Interpretability refers to models that are transparent by construction, allowing clinicians to directly trace how inputs are transformed into outputs. Classical statistical models such as linear regression and decision trees exemplify this paradigm. In oncological applications, interpretable models may incorporate physics-based constraints or biologically grounded assumptions to preserve alignment with established disease mechanisms. However, such transparency typically limits representational capacity, limiting performance in high-dimensional imaging domains where complex spatial and textural interactions predominate.

Explainability, in contrast, applies to post hoc techniques designed to interrogate complex models particularly deep neural networks whose internal reasoning is not inherently transparent. Post hoc architectures contain millions of nonlinear parameters, rendering direct inspection impractical; consequently, exploratory transparency techniques are employed to support interpretability. Methods such as saliency maps and feature attribution provide provisional approximations of the spatial regions influencing a prediction. This distinction formalizes structural tension: while deep learning systems frequently achieve superior predictive accuracy in oncological imaging, their opacity necessitates auxiliary explanation mechanisms to support accountability and safe clinical use. Methods such as saliency maps and Grad-CAM++ approximate which regions influence predictions (see Section 4.1.1 for technical details).

The demand for explainability extends beyond technical curiosity; it is central to clinical validation and safety. In practice, clinicians must determine whether algorithmic predictions are grounded in meaningful pathological features rather than spurious correlations such as scanner artifacts, demographic imbalances, or institutional biases. Without explanatory insight, models risk being correct for nongeneralizable reasons, undermining reliability across settings.

Ethically, transparency underpins professional accountability. Clinical decision-making requires justifiable reasoning, and physicians remain responsible for diagnostic and therapeutic outcomes. Opaque AI systems complicate this obligation, as clinicians cannot reasonably defend or contest recommendations that lack intelligible rationales. Furthermore, limited transparency increases susceptibility to automation bias, whereby users defer excessively to algorithmic outputs despite uncertainty. Explainability also intersects with patient autonomy and informed consent, particularly in high-stakes contexts such as cancer staging and treatment planning, where understanding the basis of recommendations is ethical.

Regulatory frameworks increasingly formalize these expectations. The U.S. Food and Drug Administration’s Action Plan for AI/ML-based Software as a Medical Device (SaMD) emphasizes transparent documentation of model behavior and performance boundaries, whereas the European Union’s General Data Protection Regulation (GDPR) articulates a “right to explanation” for consequential automated decisions [17]. Failure to meet these standards not only weakens clinical trust but also may impede regulatory approval and deployment.

Operationally, explainable artificial intelligence enhances diagnostic verification and workflow efficiency. In imaging contexts such as breast, lung, and prostate cancer, explanation tools allow clinicians to confirm that model attention corresponds to established radiographic indicators including microcalcifications, irregular lesion margins, or spiculated borders rather than artefactual cues [43,92,93,94]. Techniques such as Grad-CAM++ generate spatial heatmaps over ultrasound or MR images, enabling radiologists to assess alignment between model saliency and pathological structures of interest. This capability is particularly valuable in ambiguous cases, where AI must augment rather than override clinical judgement.

Explainability also supports workflow optimization. AI-driven triage systems (CADt) can prioritize urgent findings such as pneumothorax or pleural effusions within radiology worklists. In chest X-ray interpretation, such systems reduce reading times by approximately 35.81%, facilitating faster clinical response while preserving safety oversight [95]. Visual explanations enable rapid confirmation of AI-flagged abnormalities, mitigating cognitive burden and reducing unnecessary manual review.

In precision oncology, explainability links imaging phenotypes to therapeutic decision-making. Radiomic models can associate imaging features with genomic alterations to predict treatment response and disease progression. In radiation oncology, interpretable segmentation frameworks assist in dose optimization by highlighting tumour boundaries and organs at risk (OARs). Similarly, in image-guided interventions such as prostate brachytherapy, real-time explanatory guidance enhances targeting accuracy [93]. Across modalities including lung, breast, prostate, and dermatologic imaging, transparent attribution mechanisms facilitate domain-specific validation, ensuring that predictions remain grounded in clinically relevant morphology rather than confounding artifacts.

Despite these benefits, explainability does not eliminate epistemic uncertainty. While this exploratory transparency layer allows clinicians to check if model attention matches radiographic indicators, it does not guarantee causal fidelity or robustness. As such, explainability should be understood as one component of a broader trust framework complementing data integrity, generalizability, fairness, and uncertainty quantification.

Accordingly, the subsequent sections examine data-centric and system-level barriers that further constrain the reliability and generalizability of deep learning based oncology systems, shifting the focus from interpretability mechanisms to the structural determinants of trustworthy AI in clinical deployment.

### 3.2. Data-Centric Barriers to Reliability

The clinical translation of deep learning systems in oncology from experimental prototypes to reliable diagnostic tools remains fundamentally constrained by the quality, structure, and representativeness of the data on which these models are trained. While architectural advances such as Vision Transformers and large-scale foundation models have substantially improved predictive performance, they have also revealed a persistent “trust gap” rooted in data-related limitations rather than model design alone [96,97]. In clinical oncology, trust is a multidimensional construct that depends on robustness to noise, fairness across patient populations, and consistent performance across institutions. Given the biological heterogeneity of cancer, challenges such as label noise, annotation scarcity, hidden stratification, and institutional bias remain central barriers to explainable and generalizable AI systems [98].

#### 3.2.1. Label Integrity Crisis

Supervised deep learning assumes that training labels represent an objective ground truth. However, in oncology, diagnostic labels often reflect subjective interpretations rather than absolute biological certainty. This ambiguity introduces label noise, which can substantially degrade model reliability [99]. Deep neural networks are known to exhibit a memorization effect, whereby they initially learn meaningful signals but eventually fit noise in the training data, leading to deceptively high internal performance that fails to generalize clinically [100].

Label corruption arises from both biological ambiguity and human variability. Interobserver variability reflects disagreement among experts due to differences in training, experience, or diagnostic thresholds, resulting in inconsistent ground-truth annotations. In contrast, intraobserver variability captures inconsistencies within the same expert over time, often driven by fatigue or cognitive bias. Together, these effects introduce stochastic noise that undermines model stability and reproducibility. Mathematically, such noise is often modelled using a transition matrix $\tau \in R^{k\times k}$, where each element $\tau_{ij}$ represents the probability that a true label i is observed as a label j [100]. While symmetric noise assumes uniform corruption, oncology datasets more commonly exhibit asymmetric noise, where certain pathological states, such as high-grade dysplasia and invasive malignancy, are systematically confused. This phenomenon poses a direct threat to explainability. When a model learns from corrupted labels, attribution maps may highlight clinically irrelevant regions, creating a misleading appearance of interpretability.

To mitigate these effects, recent state-of-the-art approaches focus on robust learning strategies. Coteaching and peer-learning frameworks train paired networks that identify and exclude high-loss samples, reducing the influence of noisy labels [101]. Complementary approaches include robust loss functions such as generalized cross entropy and categorical focal loss, which downweigh outliers and reduce sensitivity to mislabelled instances [102]. Transition-matrix estimation techniques further attempt to model instance-dependent noise linked to image characteristics, whereas label-smoothing and warm-up strategies such as those used in MedSSL limit early overfitting to corrupted labels. Despite these advances, persistent uncertainty motivates the use of active learning frameworks in which models defer ambiguous cases to expert review, reinforcing human oversight in high-risk decisions.

#### 3.2.2. Annotation Bottleneck

Beyond label noise, the scarcity and cost of expert annotation represent major bottlenecks in clinical AI development. High-quality oncological annotations require specialized medical expertise, making large-scale supervision prohibitively expensive. For example, manual segmentation of complex tumours such as pancreatic cancers can take more than one hour per case, rendering conventional supervised learning economically and logistically infeasible at scale [103]. This challenge is compounded by protocol drift, where annotation standards vary across institutions, further undermining dataset consistency and model generalizability.

To address this limitation, the field has increasingly shifted toward foundation models and self-supervised learning (SSL). These approaches leverage large volumes of unlabelled or weakly labelled data to learn generalizable representations before task-specific fine-tuning. Notable examples include MedSAM, which enables universal segmentation across dozens of cancer types [104], and multimodal models such as LLaVA-Med, which integrate vision and language for clinical reasoning [105]. Radiology-specific foundation models have also demonstrated superior performance over general-purpose systems such as GPT-4 V in report generation tasks [106]. However, these models introduce new risks, including hallucination where outputs appear plausible but are clinically incorrect posing a significant challenge to trust and safety [107].

Semisupervised learning further bridges the gap between data abundance and expert scarcity. Techniques such as consistency regularization and pseudo labelling enable models to learn from unlabelled data by enforcing prediction stability under perturbations [108,109]. Frameworks such as the VESSA combine visual and semantic representations to improve boundary delineation and achieve performance comparable to that of fully supervised models while requiring substantially fewer expert annotations [104].

#### 3.2.3. Imbalanced Datasets

Oncological datasets are frequently dominated by common disease subtypes, leading to class imbalance that biases model performance toward majority classes. As a result, rare but clinically critical conditions are often underrepresented, reducing diagnostic sensitivity where it is most needed [110]. A particularly insidious manifestation of this problem is hidden stratification, in which unlabelled subgroups exist within a broader diagnostic category. In such cases, models may achieve high overall performance metrics while failing catastrophically on clinically important subpopulations. For example, in a study characterizing hidden stratification across both the CIFAR-100 benchmark and several medical imaging datasets (including chest, pelvic, and musculoskeletal radiographs), it was observed that models can exhibit relative performance differences of over 20% on clinically critical subsets such as rare but aggressive cancer subtypes despite having high aggregate AUC values. These disparities are typically found in unidentified strata characterized by low prevalence, subtle discriminative features, or spurious correlates [111]. This false sense of reliability promotes shortcut learning and masks inequities in model behaviour.

Generative data augmentation has emerged as a potential mitigation strategy. Denoising diffusion probability models (DDPMs) now outperform traditional GANs in synthesizing high-fidelity medical images, particularly for rare cancer phenotypes [112]. Techniques such as DreamBooth and latent diffusion enable the controlled generation of rare lesion appearances, expanding dataset diversity [113,114]. However, for such synthetic data to be clinically useful, they must preserve biomarker integrity, ensuring that the data maintain the subtle texture gradients required for diagnostic accuracy [115].

#### 3.2.4. Dataset Shift

A final and pervasive challenge in clinical AI is dataset shift; whereby model performance degrades when deployed outside the training environment. For instance, a systematic review of 86 deep-learning algorithms in radiology found that 81% of models exhibited decreased accuracy on external datasets, with nearly 25% experiencing a substantial performance drop of ≥0.1 in Area Under Curve (AUC). This degradation is primarily attributed to domain shifts, which occur due to variations in scanner hardware, imaging protocols, and patient demographics such as when a model trained on standard datasets is applied to an elderly trauma population or across different racial groups [116]. These shifts arise from variations in scanner hardware, imaging protocols, patient demographics, and evolving clinical practices. Such discrepancies often expose shortcut learning, where models rely on nonbiological correlates such as scanner-specific artifacts or embedded metadata rather than true pathological features [117]. To quantify and mitigate these effects, auditing frameworks such as G-AUDIT have been developed to detect and correct spurious correlations linked to institutional bias [118].

Federated learning (FL) has also emerged as a promising strategy for cross-institutional training without centralizing sensitive data. However, FL introduces its own challenges, including statistical heterogeneity due to non-IID data distributions and performance degradation caused by privacy-preserving mechanisms such as differential privacy [119,120]. Recent studies suggest that harmonized preprocessing pipelines such as standardized region extraction in breast imaging can significantly reduce intersite variability, improving federated model performance by up to 35% and narrowing the gap with centrally trained systems [119].

Taken together, these data-centric limitations reveal that reliability in oncological AI is not solely a function of model architecture but of the informational substrate on which models are trained. Label corruption, annotation scarcity, class imbalance, and institutional bias do not operate in isolation; rather, they interact to produce fragile representations that are highly sensitive to distributional variation and hidden confounders. When such systems are deployed beyond their original training environments, these compounded data deficiencies manifest as degraded robustness, inequitable performance across subpopulations, and vulnerability to spurious correlations. Consequently, addressing data integrity is a foundational prerequisite for achieving the robustness, fairness, and generalization required for safe clinical translation.

### 3.3. Robustness, Fairness, and Generalization

In oncological artificial intelligence (AI), robustness denotes a model’s ability to maintain stable and reliable performance under clinically realistic variability. As outlined in Section 3.2, however, data-centric limitations often produce representations that are brittle rather than invariant. When systems trained on homogeneous datasets are deployed across heterogeneous clinical environments, variations in scanner hardware, acquisition protocols, contrast agents, and patient demographics expose their reliance on superficial statistical regularities. Segmentation models optimized for T2-weighted MR images, for example, frequently fail when applied to T1-weighted sequences or scans with altered slice thickness, reflecting sensitivity to pixel-level intensity distributions rather than stable anatomical abstractions.

Such failures are frequently rooted in hardware-induced intensity heterogeneity. Manufacturer-specific imaging signatures arising from platforms such as Siemens or Hologic generate systematic differences in pixel distributions to which convolutional neural networks inadvertently overfit, despite identical underlying pathology [121]. These dependencies reveal a core limitation of contemporary deep learning: optimization for predictive likelihood does not guarantee the acquisition of semantically meaningful or causally grounded representations.

To mitigate cross-domain instability, research has increasingly focused on domain adaptation (DA) and domain generalization (DG). Unsupervised domain adaptation methods align feature distributions between source and target domains without requiring labelled target data, often through maximum mean discrepancy (MMD) minimization or adversarial feature alignment. The domain shift resilient mammography classification (DoSReMC) framework exemplifies this approach by identifying batch normalization layers as a major source of scanner sensitivity. By recalibrating only batch normalization and fully connected layers while freezing convolutional filters, DoSReMC enables efficient adaptation to new imaging environments without full retraining, thereby improving cross-site generalizability with limited computational overhead.

#### 3.3.1. Multicentre Variability

The transition from Tier 1 internal validation to Tier 2 multicentre, external validation frequently exposes a pronounced ‘generalization crisis’, where models optimized for specific institutional datasets fail to maintain stability across different scanner vendors. Models often learn nonbiological shortcuts such as scanner noise patterns or acquisition artifacts rather than true pathological features. This phenomenon is illustrated by a study of 107 patients and 204 lesions across three healthcare centres in the Netherlands, which evaluated the classification of high-grade vs. low-grade prostate cancer. In this multi-vendor environment involving GE, Philips, and Siemens scanners, radiomics models that achieved a mean internal AUC of 0.75 saw their performance decrease to 0.54 when applied to external, unseen datasets. This ‘generalization crisis’ highlights that models performing well in a single-centre setting may not remain reliable when faced with the technical variability of different clinical sites [122]. Although absolute human performance in such complex tasks may be modest, clinician diagnostic accuracy typically remains relatively stable across institutions, underscoring a disparity in cross-site resilience between humans and algorithms.

Hardware configurations further amplify these discrepancies. The use of endorectal coils (ERC) in MRI, for instance, alters local magnetic fields and introduces device-specific artifacts. Human experts cognitively contextualize such distortions, whereas deep learning systems frequently internalize them as predictive features. Emerging solutions aim to explicitly encode acquisition variability. The “Protocol Genome” framework, for example, extracts embeddings from Digital Imaging and Communications in Medicine (DICOM) metadata to model protocol-level heterogeneity and reduce spurious correlations across institutions [123]. These approaches reflect a growing recognition that robustness requires modelling, rather than ignoring, sources of variation.

#### 3.3.2. Adversarial Vulnerability

Beyond distributional variability, oncological AI systems demonstrate marked vulnerability to adversarial perturbations—subtle, often imperceptible input modifications that induce incorrect predictions. Compared with models trained on natural images, medical imaging systems exhibit substantially lower algorithmic stability. Empirical analyses indicate that perturbations as small as ε = 0.004 can reduce classification accuracy to 25.6% for CT, 23.9% for mammography, and 6.4% for MRI [124]. These results suggest that models rely heavily on high-frequency statistical patterns that are imperceptible to clinicians but easily disrupted computationally.

Although adversarial training can partially mitigate such fragility, it rarely restores baseline performance and often introduces a trade-off between robustness and overall accuracy. This tension highlights a structural limitation of contemporary deep learning systems: they optimize statistical correlation rather than causal or semantically grounded representations. In safety-critical oncological contexts, such instability challenges assumptions of reliability and highlights the need for robustness as a primary design objective rather than a post hoc adjustment.

#### 3.3.3. Algorithm Fairness and Demographic Equity

Robustness in clinical AI extends beyond technical stability to encompass fairness and demographic equity. Recent evidence has demonstrated that deep learning models can infer sensitive attributes such as race from medical images with high accuracy (AUC 0.91–0.99), even in the absence of explicit racial markers [125]. This capacity raises significant ethical concerns, as models may implicitly encode demographic proxies and apply uneven diagnostic thresholds across patient groups.

Approximately 29.3% of pathological AI systems exhibit performance disparities across sex, race, or age categories [126]. Methods such as the FAIR-Path framework employ contrastive learning strategies to suppress demographic proxies, reducing disparity while preserving diagnostic accuracy. Nevertheless, dataset imbalance remains a structural barrier. Rare but clinically aggressive subtypes such as triple-negative breast cancer are frequently underrepresented, transforming hidden stratification from a statistical concern into an ethical one when high-risk subpopulations experience systematic performance degradation.

These risks are compounded by the “biased ruler” effect, wherein models trained on imperfect or historically biased annotations inherit and amplify existing diagnostic inequities [127]. Age-related physiological variation, including increased breast density in younger patients, introduces additional confounders that further reduce fairness in segmentation and detection tasks. Despite these concerns, demographic transparency remains limited; approximately 95% of oncology AI studies omit race-related metadata, constraining meaningful bias auditing and mitigation [128].

Addressing these challenges demands more than incremental architectural refinement. It requires explicit modelling of domain variability, adversarial resilience, and demographic equity, along with rigorous multicentre validation and transparent reporting. Without such safeguards, improvements in internal accuracy risk masking brittle and inequitable systems that fail under real-world conditions. Only by integrating robustness, fairness, and generalization as coequal design objectives can oncological AI achieve the level of trust required for safe and ethical clinical deployment.

### 3.4. Failure Modes of Explainability Methods

Explainable artificial intelligence is frequently proposed as a remedy for concerns surrounding robustness, fairness, and generalization in deep learning systems. However, despite its growing prominence in oncological AI, current explainability approaches exhibit substantial theoretical and practical limitations that constrain their reliability in high-stakes clinical decision-making. As deployment exposes models to distributional instability and demographic inequity (Section 3.3), post hoc explanations are often relied upon to restore confidence and transparency. However, instability, methodological inconsistency, limited fidelity to underlying model logic, and insufficient clinical validation collectively undermine the epistemic security that such tools are intended to provide. Rather than fully resolving the “black-box” problem, many explainability techniques risk introducing additional layers of interpretive uncertainty.

#### 3.4.1. Instability and Technical Inconsistency of Explanations

A fundamental limitation of widely used post hoc techniques such as local interpretable model-agnostic explanations (LIME), Shapley additive explanations (SHAP), and gradient-weighted class activation mapping (Grad-CAM) is their inherent instability (see Section 4 for technical details). Empirical evidence indicates that minor, clinically insignificant perturbations to input data can produce substantial changes in explanation outputs, even when the model’s prediction remains unchanged. In medical imaging settings, explanation stability has been reported to decrease by as much as 53% following the introduction of only 10% input noise [9].

This sensitivity suggests that post hoc explanations may reflect artifacts of the input distribution rather than stable, semantically grounded decision processes. Compounding this limitation is the “disagreement problem,” wherein different explainability methods generate conflicting rationales for the same prediction. In the absence of a ground-truth explanation, clinicians lack a principled basis for adjudicating between competing visualizations. Such inconsistency challenges the assumption that post hoc explanations faithfully represent internal model reasoning, particularly in safety-critical domains such as oncology.

#### 3.4.2. Misleading Visual Explanations and Shortcut Learning

Saliency-based visual explanations are often perceived as intuitive and clinically interpretable. However, this apparent transparency can be deceptive. Heatmap-based methods are prone to highlighting spurious or noncausal features including surgical staples, skin markings, pen annotations, or scanner-specific artifacts that correlate with disease labels in training data but lack pathological relevance. This behaviour exemplifies how technical fragility in explainability methods directly contributes to the evaluative limitations discussed in Section 5.4.1, where shortcut learning can falsely inflate accuracy benchmarks by relying on non-clinical cues.

Critically, saliency maps rarely distinguish between causal drivers of a prediction and correlated byproducts of the data distribution. As a result, clinicians may infer mechanistic insight where none exist. This “illusion of interpretability” risks obscuring underlying model fragility, particularly when explanations visually align with clinical intuition. In deployment settings with limited external validation, such persuasive but unreliable visualizations may inadvertently amplify misplaced trust rather than mitigate it.

#### 3.4.3. Limitations to Fidelity and the Risk of False Confidence

Beyond instability and visual ambiguity lies a deeper structural concern: the fidelity gap between explanations and the underlying model. Fidelity (or faithfulness) is defined as the degree to which an explanation accurately reflects the model’s internal decision-making process, as opposed to merely appearing plausible to a human observer. Many post hoc techniques approximate complex, high-dimensional decision boundaries using simplified local surrogate models. While these approximations may appear internally coherent, they do not necessarily reproduce the true global behavior of the original network. Consequently, explanations can be self-consistent yet misaligned with the model’s actual decision logic.

This misalignment poses a significant risk of false reassurance. Visually intuitive overlays or feature attributions may create the impression of transparency without guaranteeing epistemic validity. In clinical contexts, such misplaced confidence may bias practitioners toward AI-supported recommendations, even when contradictory clinical evidence is present. In the absence of formal mechanisms to quantify explanation reliability or uncertainty, explainability tools may inadvertently reinforce, rather than attenuate, diagnostic risk.

#### 3.4.4. Clinical Validation Gap

A further limitation of current XAI research is the absence of standardized, clinically grounded evaluation frameworks. Most interpretability methods are assessed using technical proxies such as sparsity, faithfulness, or completeness that lack direct correlation with clinical utility. Consequently, a substantial gap persists between algorithmic explainability and real-world interpretability in oncological practice.

Prospective evaluations examining whether explainability improves diagnostic accuracy, reduces error rates, or enhances treatment decisions remain scarce. Few studies have demonstrated measurable downstream benefits, such as improved patient outcomes or safer therapeutic choices. Without such evidence, XAI remains largely a conceptual safeguard rather than an operationally validated solution. Addressing this disconnect requires interdisciplinary evaluation protocols, domain-specific benchmarks, and prospective clinical trials capable of defining what constitutes a clinically meaningful explanation.

In summary, while explainable AI represents an essential component of trustworthy medical AI, current approaches remain constrained by instability, ambiguity, limited fidelity, and insufficient clinical validation. Overcoming these limitations demands a shift from visually persuasive post hoc rationalizations toward causally grounded, uncertainty-aware, and clinically validated interpretability frameworks that substantively support human decision-making rather than merely accompany it. Table 2 provides a comprehensive summary of the system-level barriers encountered across the deep learning development–deployment pipeline in oncological imaging, detailing specific technical constraints, trade-offs, and their corresponding clinical implications

## 4. Explainable AI in Cancer Imaging: Methods and Clinical Alignment

To systematically examine explainability strategies in oncology imaging, it is useful to categorize existing approaches according to their underlying design philosophy and level of interpretability. Broadly, explainability methods can be grouped into post hoc techniques, intrinsically interpretable architectures, and human-centred explanation frameworks.

The following subsections examine each category in detail, beginning with post hoc explanation methods and progressing toward intrinsically interpretable and clinician-centred approaches. To provide a structured comparison of the major explainable AI approaches used in oncology imaging, their underlying principles, strengths, limitations, and clinical applicability are summarized in Table 3.

### 4.1. Post Hoc Explainability Approaches

Post hoc explainability encompasses a collection of techniques designed to interpret the decision-making processes of deep learning models after training has been completed [129]. These approaches are particularly relevant in oncological imaging, where high-performing architectures such as deep convolutional neural networks and vision transformers (ViTs) are frequently deployed despite their inherent opacity [130]. Rather than embedding interpretability constraints directly into model design, post hoc methods seek to translate abstract internal representations into clinically interpretable evidence.

In practice, these techniques generate visual or quantitative explanations, most commonly heatmaps superimposed on mammograms, CT scans, MRI, or histopathology slides—to indicate image regions that most strongly influence model predictions. Broadly, post hoc methods can be categorized into three principal groups: attribution-based (gradient-driven), perturbation-based (model-agnostic), and attention-based approaches.

While methods such as Grad-CAM, LIME, and SHAP are widely adopted, they should be regarded as exploratory tools that provide proxy insights rather than definitive explanations. These tools function as exploratory approaches to transparency, inherently constrained by limited fidelity and the absence of causal grounding and should be considered methodologically limited techniques under ongoing scrutiny rather than definitive standards. From the following subsections, we realize that these methods do not yet meet the threshold for clinical decision relevance or trust calibration.

#### 4.1.1. Gradient-Based and Saliency Mapping Techniques

Gradient-based methods constitute a foundational class of post hoc explainability techniques, offering computationally efficient visualization of class-specific relevance. While earlier sections of this review utilized Grad-CAM as a conceptual example of post hoc explanation and its associated clinical barriers, this section provides the formal methodological foundation and mathematical analysis of gradient-based saliency mapping. Early work introduced class activation mapping (CAM), which computes a weighted sum of the final convolutional feature maps using weights derived from the global average pooling (GAP) layer [131]. However, CAM requires architectural modifications, specifically the presence of a GAP layer preceding the softmax output, thereby limiting its applicability to pretrained models [132].

To address this constraint, Grad-CAM was developed. Grad-CAM computes the gradient of the target class score *y^c^* with respect to feature maps *A^k^* from the final convolutional layer. The importance of the weight for feature map *k* is defined as follows:

(1) $$\alpha_{k}^{c}=\frac{1}{Z}\sum_{i}\sum_{j}\frac{\partial y^{c}}{\partial A_{ij}^{k}},\;$$

where $A_{ij}^{k}$ denotes the activation at spatial location (*i*,*j*) in the *k^th^* feature map and *Z* represents the total number of spatial locations. The class-discriminative heatmap is then obtained via a weighted sum of the feature maps followed by a rectified linear unit (ReLU), ensuring that only features positively contributing to the target class are retained [132].

Grad-CAM is architecture-agnostic and is a widely adopted architecture-agnostic tool, frequently critiqued for coarse localization and an inability to accurately represent multiple lesions or irregular, complex tumour boundaries [133].

Grad-CAM++ extends the original Grad-CAM framework by introducing a more rigorous weighting formulation that leverages higher-order gradient information to improve localization accuracy. Specifically, Grad-CAM++ incorporates second order (and implicitly higher-order) partial derivatives of the target class score with respect to feature map activations to compute pixelwise importance weights. Unlike Grad-CAM, which assigns a single global weight to each feature map via spatially averaged first-order gradients, Grad-CAM++ derives adaptive weights that account for the contribution of each spatial location within the feature maps. This formulation enables a more precise attribution of class-specific importance, particularly in cases where multiple instances of the target object are present or when the regions of interest exhibit complex spatial distributions.

By integrating pixel-level weighting with higher-order gradient sensitivity, Grad-CAM++ produces sharper and more complete activation maps, thereby enhancing localization fidelity. This is especially advantageous in oncology imaging, where tumours may appear as multiple lesions or exhibit heterogeneous and diffuse boundaries. In such scenarios, the improved sensitivity of Grad-CAM++ to distributed evidence allows for more comprehensive identification of clinically relevant regions compared to standard Grad-CAM. However, like other advanced gradient-based techniques, the incorporation of higher-order derivatives introduces additional computational complexity, which may impact scalability in large-scale or time-sensitive clinical workflows.

Building upon these advances, Guided Grad-CAM further enhances interpretability by integrating the coarse class-discriminative localization of Grad-CAM with the high-resolution detail of guided backpropagation. Specifically, Guided Grad-CAM is obtained through element-wise fusion of the Grad-CAM heatmap with guided backpropagation gradients, thereby combining spatially localized class relevance with fine-grained pixel-level sensitivity. While Grad-CAM effectively highlights the region’s most influential for a given prediction, its spatial resolution is inherently limited by the underlying feature maps. In contrast, guided backpropagation captures high-frequency gradient information but lacks class specificity. Their fusion enables the generation of saliency maps that are both class-discriminative and visually detailed.

This hybrid representation yields enhanced delineation of morphological structures, making it particularly well-suited for oncology imaging applications that require fine-grained tissue characterization. In digital pathology, for instance, such detailed visualizations can facilitate the identification of subtle histomorphological patterns associated with underlying genetic alterations or molecular subtypes. By preserving both localization fidelity and structural detail, Guided Grad-CAM provides a more comprehensive interpretability framework, bridging the gap between coarse regional attribution and pixel-level explanation [134].

Nevertheless, it is important to note that Guided Grad-CAM inherits certain limitations from its constituent methods, including sensitivity to gradient saturation and potential susceptibility to noise in high-frequency components. Additionally, the combined computation may introduce moderate overhead compared to standard Grad-CAM, although it remains more efficient than fully sampling-based approaches.

SmoothGrad extends conventional gradient-based saliency methods by addressing the inherent noise and instability in raw gradient visualizations. Instead of relying on a single gradient map, SmoothGrad generates multiple saliency maps by introducing Gaussian noise to the input image and computing gradients for each perturbed instance. These individual maps are then averaged to produce a final, smoothed saliency representation. This process effectively suppresses high frequency “gradient noise” that often arises due to local fluctuations in model sensitivity, leading to sharper and more spatially coherent importance regions. As a result, SmoothGrad improves the stability and robustness of visual explanations, particularly in high-dimensional medical imaging contexts such as oncology, where subtle pixel-level variations can significantly influence model predictions.

However, this enhanced interpretability comes at the cost of increased computational overhead, as multiple forward and backward passes are required to generate and aggregate the perturbed saliency maps. Despite this trade-off, the improved reliability and consistency of explanations make SmoothGrad and related noise-averaging techniques valuable complements to methods such as Grad-CAM, especially in clinically sensitive applications where interpretability must be both precise and dependable.

While gradient-based methods are efficient and visually intuitive, their reliability remains sensitive to model architecture, input perturbations, and gradient saturation effects.

#### 4.1.2. Perturbation-Based and Model-Agnostic Approaches

Perturbation-based techniques treat predictive models as black boxes and interrogate behavior by systematically modifying input features. Among the most widely adopted approaches are local interpretable model-agnostic explanations (LIME) and Shapley additive explanations (SHAP).

LIME approximates the complex decision boundary of a model with a simpler, interpretable surrogate (typically linear) within the local neighborhood of a specific instance [135,136]. In clinical imaging contexts, LIME has demonstrated strong local fidelity, with meta-analytic evidence in radiology reporting average fidelity scores of 0.81, compared with 0.38 for SHAP and 0.54 for Grad-CAM [9]. Nevertheless, LIME is sensitive to segmentation granularity and the number of perturbations applied, which may introduce instability and reduce reproducibility across repeated runs.

SHAP, which is grounded in cooperative game theory, assigns feature importance values based on each feature’s marginal contribution to the model’s prediction across all possible subsets [136,137,138]. This theoretical consistency makes SHAP particularly suitable for multimodal frameworks that integrate imaging with genomic or structured clinical data. SHAP provides both local explanations (e.g., force plots) and global summaries of feature importance. However, exact computation of Shapley values is computationally intractable because of the need for exponential feature combinations, which require approximation methods for practical implementation.

Although perturbation-based approaches offer stronger theoretical grounding than gradient methods do, they are computationally intensive and may struggle with high-dimensional pixel-level representations typical of medical imaging.

#### 4.1.3. Attention Visualization in Vision Transformers

The increasing adoption of vision transformers (ViTs) introduces a distinct paradigm for interpretability via multihead self-attention (MSA) mechanisms. Unlike CNNs, which rely on local receptive fields, ViTs model long-range spatial dependencies through interactions among query (*Q*), key (*K*), and value (*V*) vectors. The self-attention operation is defined as follows:

(2) $$\mathrm{Attention}\left(Q,K,V\right)=\mathrm{Softmax}\left(\frac{QK^{⊤}}{\sqrt{d_{k}}}\right)V,$$

where d_k_ denotes the dimensionality of the key vectors [139].

In digital pathology, this mechanism enables the identification of diagnostically relevant regions within whole-slide images without pixel-level annotations [140]. Attention maps can serve as intuitive visual explanations by highlighting regions that contribute most strongly to predictions. Some frameworks further personalize attention patterns by incorporating patient-specific metadata and aligning model focus with clinically relevant contextual factors [140].

ViTs have also demonstrated enhanced robustness relative to CNNs in low-quality histopathological settings, maintaining superior performance in tasks such as margin assessment for squamous cell carcinoma despite imaging artefacts [91]. However, whether attention weights constitute faithful explanations of causal reasoning remains debated, as attention visualization does not necessarily guarantee mechanistic transparency.

Post hoc explainability methods provide retrospective insights into model decision-making. Table 4 summarizes prominent approaches in oncology imaging, highlighting their strengths, clinical alignment, and limitations. The table organizes studies according to model type, explainability technique, validation strategy, interpretability metrics, and observed limitations. The table synthesis addresses Research Question 2 (RQ2) regarding the capacity of XAI methods to enhance clinician trust and provide the foundational evidence required for diagnostic validation. The compilation also helps in addressing Research Question 3 (RQ3) by examining the specific deep learning techniques and architectural modifications that improve the intrinsic interpretability of cancer image classifiers.

These results suggest that while post hoc methods can visually highlight diagnostically relevant regions, they are sometimes limited by coarse localization, gradient noise, or the inability to fully reflect clinical reasoning. Selecting task-specific techniques and combining them with domain knowledge can enhance trust and usability.

From this summary, it is evident that gradient-based methods (e.g., Grad-CAM, Grad-CAM++) and perturbation-based approaches (e.g., LIME, SHAP) are widely adopted for visual and quantitative explanations. However, these methods often face limitations in terms of clinical fidelity, computational cost, and semantic alignment with expert reasoning. The insights from this table set the stage for comparing intrinsic and hybrid models, which aim to overcome some of these post hoc constraints.

### 4.2. Intrinsic and Hybrid Interpretable Models

The transition from provisional post hoc approximations toward intrinsically interpretable architectures such as concept bottleneck models (CBMs) is driven by the need to resolve the fidelity and validation crises inherent in saliency-based tools. Unlike Grad-CAM, which approximates ‘where’ a model looks, intrinsic models provide transparency by design, ensuring that reasoning is semantically anchored in medical ontologies from the outset.

Intrinsic explainability refers to modelling architectures whose decision-making processes are structurally interpretable without reliance on post hoc analysis [129]. In contrast to attribution-based explanations that approximate internal reasoning after inference, intrinsic models embed interpretability directly into their computational structure. Within oncological imaging, this paradigm shifts from opaque latent embeddings toward intermediate representations that encode clinically meaningful concepts, prototypical examples, or biologically constrained relationships.

The clinical value of intrinsic models lies in their alignment with established medical ontologies and reporting standards, including the Breast Imaging Reporting and Data System (BI-RADS) and the tumour–node–metastasis staging system [141]. By structuring predictions around recognizable descriptors such as lesion margins, calcification patterns, or nodal involvement these architectures facilitate multidisciplinary communication, enhance auditability, and enable direct verification of diagnostic reasoning. Importantly, transparency by design improves the ability to detect spurious correlations, including scanner-specific artifacts or institutional biases, before clinical deployment [142].

Rather than forming a single methodological class, intrinsic and hybrid interpretable models have evolved along four interrelated directions: (i) concept-bottleneck architectures, (ii) prototype-based reasoning, (iii) biologically constrained and disentangled representations, and (iv) neurosymbolic hybrids.

#### 4.2.1. Concept Bottleneck and Clinically Structured Architectures

Concept Bottleneck Models represent among the most direct approaches to clinical alignment [143]. These models decompose prediction into two sequential mappings: a concept predictor g, which transforms raw inputs into a vector of human-interpretable concept probabilities, and a label predictor f, which generates the final diagnosis exclusively from these concepts. Formally:

(3) $$\hat{y}=f\left(g\left(x\right)\right),g\left(x\right)\in R^{K}$$

where *g*(*x*) corresponds to predicted clinical attributes (e.g., “spiculated margin” and “heterogeneous enhancement”) and f is typically constrained to a linear or monotonic form to ensure transparency [143].

This explicit decomposition enables clinicians to determine whether malignancy predictions are supported by appropriate morphological evidence. CBMs have been applied in kidney cancer survival modelling using TNM-aligned graph neural network concepts [57] and in hematological imaging tasks where contrastive objectives capture interpretable cellular features such as granule density.

A key advantage of CBMs is controllability: During inference, clinicians may correct erroneous concept prediction, and the final output is updated accordingly, enabling human-in-the-loop refinement [144]. However, conventional CBMs impose substantial annotation burdens, requiring concept-level supervision alongside diagnostic labels [143].

Recent developments mitigate this limitation. Knowledge-enhanced bottleneck models incorporate priors derived from medical literature, whereas methods such as MONET and concept multiple instance learning leverage pretrained vision language models to infer clinical concepts without exhaustive manual labelling [145,146,147]. Self-supervised approaches, including E-BotCL, further reduce supervision requirements by discovering task-relevant concepts through dual-path contrastive learning [148].

Despite these advances, CBMs remain susceptible to “concept leakage,” whereby noninterpretable latent features bypass the bottleneck. Quantitative transparency metrics such as the concept-task leakage (CTL) score have therefore been proposed to evaluate the degree of information isolation [149].

#### 4.2.2. Prototype-Based and Case-Based Reasoning

Prototype-based learning offers an alternative intrinsically interpretable paradigm grounded in case-based reasoning. Architectures such as ProtoPNet associate each class with a set of learned prototypical image patches that serve as reference exemplars [150].

During inference, the similarity between an input representation z and a prototype p_j_ is computed using the squared Euclidean distance in the latent space. The similarity score is defined as follows:

(4) $$S_{j}=\underset{z\in Z}{max}\;exp\left(-∥z-p_{j}{∥}_{2}^{2}\right),$$

where *Z* denotes the set of latent patch embeddings extracted from the image [151]. Because predictions are directly determined by prototype activations, explanatory evidence is causally linked to the decision, in contrast to post hoc saliency overlays that may lack semantic grounding.

Prototype-based systems have demonstrated clinical utility in mammography (e.g., IAIA-BL), where prototypes correspond to interpretable features such as mass margins, and in multiparametric MRI (e.g., MProtoNet), where soft masking attention enhances spatial coherence relative to gradient-based visualizations [74]. These models provide both global interpretability (through inspection of the learned prototype library) and local explanation (via prototype similarity for individual patients).

#### 4.2.3. Biologically Constrained and Disentangled Representations

In the third direction, domain-specific constraints are embedded directly into representation learning. Rather than predicting concepts explicitly, these architectures enforce structural properties consistent with biological or physical principles.

In survival modelling, for example, monotonic relationships between imaging biomarkers and hazard risk can be encoded through distributional constraints. The Weibull twin neural network (WTNN) embeds monotonicity into the Weibull shape parameter to ensure biologically plausible hazard trajectories [152]. Deep lattice networks similarly employ calibrated lookup tables to enforce interpretable monotonic relationships between inputs and outputs [153].

Disentangled representation learning (DRL) further separates latent variables into semantically independent components such as shape, texture, or tracer uptake intensity. The PET-Disentangler framework generates distinct latent vectors for healthy and pathological factors, enabling the synthesis of “pseudohealthy” images for comparative interpretation [154]. In federated settings, FedDis separates global structural patterns from scanner-specific artifacts, improving cross-institutional robustness without sharing raw data [155]. Unsupervised DRL systems such as MorphoGenie and DISCOVER have linked latent factors to measurable biophysical properties (e.g., DNA content), demonstrating strong empirical correlations (R ≈ 0.82) [156].

By isolating independent explanatory dimensions, disentangled models improve interpretive clarity while simultaneously mitigating domain shift.

#### 4.2.4. Neuro-Symbolic and Causality-Aware Hybrid Models

Hybrid interpretable architectures combine deep perceptual modules with structured reasoning frameworks. Neuro-symbolic systems integrate neural feature extraction with logical constraints derived from clinical knowledge bases.

Logic tensor networks encode medical axioms directly into training objectives, ensuring consistency with domain-specific rules. More recent systems, such as the Neuro-Symbolic System for Cancer (NSSC), integrate large language models to map oncological terminology from clinical documentation to the Unified Medical Language System, grounding predictions in validated ontologies [157].

Causality-aware attention mechanisms extend this paradigm. Architectures such as CausalX-Net incorporate structural causal models and counterfactual reasoning to generate causal effect maps that isolate features necessary for diagnosis, suppressing background artifacts that often confound conventional attention maps [68].

Hybrid CNN–Transformer systems, including HyFormer-Net, further integrate local texture extraction with global contextual modelling, achieving quantitative validation of attention alignment (e.g., Mean Intersection over Union ≈ 0.86) [70]. By embedding structural constraints while preserving representational power, such models aim to reconcile transparency with predictive performance.

Intrinsic and hybrid interpretable models embed transparency directly into the architecture, enabling closer alignment with clinical concepts. Table 5 provides a comparative overview of these methods and their reported clinical validations.

Table 5 summarizes intrinsically interpretable and hybrid XAI models in cancer imaging. Unlike post hoc methods, these architectures are designed for transparency by construction, leveraging concept bottlenecks, prototypes, attention-guided mechanisms, or neurosymbolic reasoning.

## 5. Human–AI Trust Calibration and Clinical Translation

The successful translation of artificial intelligence (AI) systems from experimental validation to routine oncological practice depends not solely on predictive performance but also on calibrated trust between clinicians and algorithms. In high-stakes domains such as cancer imaging, diagnostic decisions carry substantial therapeutic and ethical consequences; therefore, trust must be grounded in measurable reliability, interpretive transparency, and accountable human oversight. Technical explainability alone is insufficient if it does not translate into improved clinical judgment, stable workflow integration, and appropriately calibrated confidence in AI-assisted outputs.

Trust calibration in oncology imaging requires an integrated framework that aligns algorithmic behavior with clinical reasoning, quantifies predictive uncertainty, and embeds structured human oversight within deployment architectures. This section synthesizes these components by examining (i) human-in-the-loop (HITL) systems as mechanisms for preserving clinician agency, (ii) the limitations of accuracy-centric benchmarking in evaluating explainability, (iii) real-world human–AI interaction dynamics, and (iv) probabilistic uncertainty estimation and calibration strategies that support responsible clinical adoption. Together, these elements define the pathway from technically interpretable models to clinically trustworthy decision-support systems.

### 5.1. Multidimensional Trust: Robustness, Uncertainty, Fairness

Trustworthiness in oncological AI is not an intrinsic algorithmic property but a relational construct emerging from interactions between patients, clinicians, and AI systems across the development-to-deployment lifecycle [158]. Despite the substantial diagnostic promise of deep learning, a persistent gap in trust remains in clinical practice. Surveys indicate that approximately 83% of patients prefer human-led decision-making, citing concerns regarding legal liability, algorithmic bias, and the opaque “black-box” nature of machine reasoning [159]. In response, regulatory and policy initiatives including the European Commission’s High-Level Expert Group on AI and the FUTURE-AI framework have converged on five foundational dimensions for trustworthy clinical AI: robustness, reliability, transparency, fairness, and accountability [160].

#### 5.1.1. Robustness and Stability in Adverse Clinical Environments

Robustness denotes a model’s ability to preserve diagnostic performance under input variations that do not alter the underlying pathology [161]. In oncology imaging, deep learning systems are particularly vulnerable to adversarial perturbations—subtle pixel-level manipulations that exploit high-frequency features not typically used in human visual reasoning [124]. The clinical implications of such vulnerability are substantial. Empirical evidence has demonstrated that minimal perturbations can catastrophically degrade performance, reducing lung nodule detection accuracy from 75.4% to 25.6% and brain metastasis detection from 93.6% to as low as 6.4% [124].

Although adversarial training remains the primary defensive strategy, it introduces a robustness–accuracy trade-off, often reducing performance on nonperturbed images. Beyond adversarial threats, models must also withstand domain shifts arising from variations in scanner vendors, acquisition protocols, reconstruction kernels, or histopathological staining techniques [79]. Without robustness across such real-world variability, even highly accurate models risk unsafe deployment.

#### 5.1.2. Reliability Through Uncertainty Quantification

Clinical reliability requires not only accurate predictions but also calibrated communication of predictive confidence. Conventional deterministic neural networks fail to explicitly model aleatoric uncertainty, stemming from irreducible data noise such as motion artifacts or epistemic uncertainty, reflecting limitations in model knowledge and particularly pronounced in rare tumour subtypes.

Bayesian deep learning approaches, including Monte Carlo dropout and deep ensembles, enable stochastic estimation of predictive uncertainty [162]. In practice, uncertainty thresholding allows systems to flag the most ambiguous cases often the highest-entropy 20%, for immediate clinician review, yielding improvements exceeding 8% in binary cancer detection accuracy [163]. Spatial confidence maps further enhance uncertainty communication by visually highlighting regions of elevated ambiguity, such as poorly demarcated tumour margins in cirrhotic liver tissue. In this manner, uncertainty quantification transforms AI from a deterministic classifier into a probabilistic decision-support tool.

#### 5.1.3. Transparency and the Interpretability

Epistemic opacity remains a major barrier to the clinical adoption of deep neural networks. Although explainable AI techniques aim to mitigate this limitation, a persistent interpretability–performance paradox endures: the most accurate architectures, including Vision Transformers, are often the least transparent.

The exploratory transparency layer provided by methods such as Grad-CAM, SHAP, and LIME generates saliency maps or feature attribution scores; however, these explanations frequently exhibit limited fidelity and imperfect anatomical alignment [164]. Evidence indicates that the reliability of this exploratory transparency layer can decrease by up to 53% under clinically realistic image noise, undermining its role as a stable basis for clinical trust [9].

In response, research has shifted toward ante hoc, intrinsically interpretable architectures, such as SpikeNet, alongside the development of quantitative alignment metrics such as XAlign, which assess the concordance between model explanations and established biological markers [75]. Such approaches attempt to reconcile predictive performance with structural transparency rather than relying solely on post hoc approximation.

#### 5.1.4. Fairness, Bias Mitigation, and Accountability

Trust erodes when AI systems exhibit systematic performance disparities across demographic groups. Emerging evidence demonstrates that pathology models may inadvertently learn demographic proxies inferring attributes such as race or sex directly from histological slides and incorporate these signals into diagnostic decisions. While this capability may exceed human perceptual awareness, it introduces significant ethical and clinical risks.

Such proxy learning has been associated with reduced diagnostic accuracy in underrepresented populations, including lung cancer subtyping among African American patients, because of imbalanced genomic datasets. Mitigation strategies, including fairness-aware supervised contrastive learning frameworks such as FAIR-Path, have reduced performance disparities by up to 88.5% [126].

At the systems level, federated learning enables multi-institutional model development while preserving data privacy in compliance with the requirements of the GDPR and Health Insurance Portability and Accountability Act (HIPAA). Ultimately, accountability in clinical deployment depends on clearly defined liability structures encompassing medical malpractice, product liability, and institutional oversight, thereby situating AI within the legally recognized “reasonable physician” standard of care [165].

Together, robustness, uncertainty calibration, transparency, fairness, and accountability constitute the multidimensional foundation upon which clinician trust must be built.

### 5.2. Clinician-Centred Explainability

Clinical translation of deep learning in oncology remains constrained by a persistent “explainability gap” between high-dimensional algorithmic outputs and the heuristic, evidence-based reasoning of clinicians. Although deep learning systems have demonstrated performance comparable to or exceeding that of human experts in lesion detection and volumetric analysis, their black-box nature continues to limit adoption [166]. Clinicians require more than binary predictions; they need explanations aligned with standardized diagnostic frameworks, reduced cognitive burden, and task-specific interpretive cues appropriate for screening, grading, or staging. Interpretability must therefore achieve concordance with established clinical reasoning processes rather than merely offering mathematical transparency.

This challenge is exemplified in prostate cancer diagnostics, where the Prostate Imaging–Reporting and Data System (PI-RADS) v2.1 integrates multiparametric MRI sequences into a structured interpretive framework [167]. For clinical interpretability, model reasoning must correspond to domain-specific cues such as reliance on T2-weighted imaging for transition zone assessment and diffusion-weighted imaging for peripheral zone evaluation [168]. Evidence from the international PI-CAI challenge illustrates this distinction: although the AI system achieved an area under the receiver operating characteristic curve of 0.91, surpassing the pooled radiologist average of 0.86, it frequently failed to replicate the contextual reasoning applied in multidisciplinary clinical practice [169].

Radiologists routinely incorporate longitudinal patient history, prior biopsy results, and prostate-specific antigen levels when equivocal PI-RADS 3 lesions are resolved. Without access to such contextual information, AI models often produce clinically misaligned segmentations and risk estimates, reducing specificity during real-world deployment. To mitigate this gap, AI systems should function as structured decision support tools embedded within established diagnostic pathways rather than autonomous decision makers. For example, rule-based alerts can be triggered when clinician-entered parameters such as lesion diameter exceeding 1.5 cm mandate risk escalation. Such logic-concordant interpretability ensures that AI operates as a verification layer rather than an opaque authority [170].

To address both technical and usability challenges, a three-pillar accountability framework has been proposed, emphasizing transparent model development, interpretability-by-design architectures, and post hoc explanations augmented with explicit uncertainty indicators [9]. Central to this framework is explanation granularity, which must align with the specific clinical objective. Explainable AI approaches can be categorized into pixel-level, feature-level, and concept-level methods. While pixel-level visualizations such as Grad-CAM remain prevalent, they often provide coarse explanations insufficient for detecting subtle features such as spiculated breast lesions.

The introduction of interpretability tools also raises significant human–computer interaction challenges, particularly regarding cognitive load. Visual explanation overlays may paradoxically increase mental effort, a phenomenon described as “cognitive load exploitation,” in which interpreting the explanation becomes more demanding than the original diagnostic task. Simulation studies have shown that AI-generated heatmaps increase reporting time by approximately 33% and delay dictation by more than 40% [171]. This inefficiency is further amplified by “authority modulation,” wherein clinicians feel compelled to scrutinize all AI-highlighted regions to mitigate perceived liability risk. Eye-tracking studies measuring fixation frequency and saccade duration confirm increased cognitive strain and diminished trust among early adopters.

In digital histopathology, automated concept-based explanations translate pixel representations into clinically meaningful constructs, such as cell density or glandular architecture [172], offer greater semantic clarity. Image patches are clustered using k-means, after which testing concept activation vector (TCAV) scores quantify the influence of these concepts on model predictions. Such methods have proven effective in revealing hidden dataset biases including spurious correlations between staining protocols and cancer markers that pixelwise explanations such as guided Grad-CAM may fail to detect.

Ultimately, successful clinical integration requires a dynamic and continuously negotiated state of trust, shaped by the system’s ability to deliver clinically concordant reasoning while maintaining technical validity, safety, and usability. Trust calibration is therefore not a one-time achievement but an ongoing alignment process between algorithmic behaviour and the evolving standards of oncological care. Table 6 summarizes domain-specific requirements for clinically meaningful explanations, their alignment with decision pathways, and common failure modes.

To achieve true clinical utility, explainable AI (XAI) must move beyond general transparency toward epistemic alignment, a structural harmony between the model’s internal logic and the distinct hierarchical and contextual reasoning of oncological practice. The following synthesis analytically differentiates “clinical alignment” across oncology domains by identifying their unique interpretive units, decision pathways, and consequential failure modes.

#### 5.2.1. Breast Ultrasound: Morphological and Signal-Based Logic

In the specific context of breast ultrasound, the diagnostic logic is fundamentally morphological and signal-based, where clinical efficacy depends on the detection of subtle architectural distortions such as microcalcifications and irregular lesion boundaries that signal early-stage malignancy. Unlike more complex multiparametric modalities, the primary interpretive units in ultrasound are these discrete visual descriptors, which are codified within the Breast Imaging Reporting and Data System (BI-RADS) lexicon. For an AI system to achieve epistemic alignment in this domain, its explanatory output must move beyond simple pixel-level saliency toward semantic anchoring, grounding its predictions in these recognized clinical descriptors.

However, a significant “alignment gap” persists between current algorithmic capabilities and clinical requirements. While standard post hoc tools like Grad-CAM are widely utilized for tumour localization, they are frequently critiqued for their coarse spatial resolution and “baseline diffuseness”. Such coarse visualizations are often insufficient for the precise characterization of spiculated margins, where the difference between a benign and malignant classification may hinge on minute structural details. Furthermore, research indicates that feature attribution in these models can be highly inconsistent, with different architectures highlighting disparate regions for the same input, thereby undermining their role as a “ground truth” for medical verification.

The translational implications of these technical limitations are particularly acute in breast imaging. Breast ultrasound models exhibit a high sensitivity to visual noise and signal artefacts, which frequently leads to false positives. When explainability tools fail to distinguish between true pathology and these non-biological artefacts, they risk creating an “illusion of interpretability”. This can inadvertently foster automation bias, where clinicians over-rely on a model that is “right for the wrong reasons”, or conversely, it can increase cognitive load. In the latter scenario, described as “cognitive load exploitation”, the visual complexity of interpreting diffuse heatmaps becomes more demanding than the original diagnostic task, potentially increasing reporting times by over 33% due to “authority modulation”, the perceived need to scrutinize every AI-highlighted region to mitigate liability.

To resolve these failures, the manuscript argues for a shift toward multitask learning frameworks (e.g., BI-RADS-Net) that explicitly link visual representations to a predefined medical vocabulary. By functioning as a logic-concordant verification layer, such systems ensure that AI assistance streamlines the biopsy triage pathway by flagging only those features that align with established pathophysiological indicators. Ultimately, the goal in breast ultrasound is to transform the AI from an opaque statistical instrument into a reliable partner that provides traceable reasoning pathways aligned with the expert’s own morphological scrutiny.

#### 5.2.2. Prostate MRI: Multiparametric and Contextual Synthesis

A prostate cancer diagnosis follows a multiparametric and contextual logic, where the Prostate Imaging–Reporting and Data System (PI-RADS) v2.1 serves as the foundational interpretive framework. Efficacy in this domain requires the synthesis of diverse MRI sequences, usually balancing T2-weighted imaging for transition zone assessment against diffusion-weighted imaging (DWI) for the peripheral zone. While deep learning systems often report high technical metrics, the international PI-CAI challenge highlighted a recurring failure to replicate the contextual reasoning experts apply in practice. Specifically, radiologists integrate longitudinal patient history, previous biopsy results, and prostate-specific antigen (PSA) levels into clinical narratives that current imaging-only AI models frequently ignore. The omission of these parameters is problematic; a model operating in a vacuum, stripped of the broader clinical picture, remains “clinically misaligned” regardless of its AUC.

Technical reliability is further undermined by a “generalization crisis” stemming from scanner and vendor heterogeneity. Multi-centre data from the ProstateNet study indicates that shifts between Siemens, Philips, and GE hardware, or the use of endorectal coils (ERC), can trigger an average AUC reduction of 0.05. Deep learning systems often internalize these hardware-specific artefacts as predictive features rather than identifying stable biological abstractions. This fragility is complicated by the “disagreement problem” in post hoc explainability. When tools like Grad-CAM, LIME, and SHAP are applied to the same lesion, they often provide conflicting rationales, offering “multiple interpretations” that lack a verifiable ground truth. Such inconsistency suggests that visual heatmaps alone cannot support professional accountability or provide a stable basis for diagnostic verification. Achieving true epistemic alignment likely requires a shift toward architectures like MProtoNet, which uses prototype-based reasoning to link decisions to specific clinical exemplars, functioning as a logic-concordant verification layer rather than an opaque authority.

#### 5.2.3. Digital Pathology: Topological and Multiscale Hierarchy

Digital pathology operates through a logic of topological and multiscale hierarchy, where diagnostic significance is found not in isolated pixels but in the complex spatial organisation of the tumour microenvironment. The fundamental interpretive units in this domain are inherently hierarchical, requiring an iterative reasoning process that spans from individual cellular morphology at 20× magnification to the global architecture of entire tissue slides at 5×. Achieving epistemic alignment requires AI systems to mirror this multiscale “consultation” workflow, grounding predictions in biological constructs rather than abstract mathematical patterns.

The reliance on standard visual heatmaps in digital pathology is often a hollow victory. There is a persistent and documented risk of shortcut learning, where algorithms achieve high accuracy by mistakenly anchoring predictions on staining artefacts, pen markings, or institutional protocol drift rather than actual tumour morphology. This creates a dangerous “illusion of interpretability”; a saliency map might highlight a region that appears plausible to a human observer, but the underlying model may be exploiting non-biological correlates that fail to generalize across centres. Such technical fragility suggests that visual transparency, without structural alignment, is insufficient for professional accountability.

The extreme scale of gigapixel whole-slide imaging (WSI) further complicates this landscape, as direct end-to-end processing is computationally impractical, forcing a reliance on multiple instance learning (MIL) and intelligent sampling. Clinical utility depends on these pipelines translating raw pixel data into recognized cellular and glandular constructs. Architectures such as HACT-Net, which formalize tissue as hierarchical graphs, or Automated Concept-based Explanations (ACE), which link predictions to specific histological features, offer a more robust path forward. By forcing the AI to function as a logic-concordant verification layer, the system moves beyond being an opaque statistical instrument and begins to speak the actual language of the pathologist.

#### 5.2.4. Multimodal Oncology: Longitudinal and Integrative Reasoning

Multimodal oncology demands a fundamental shift from cross-sectional snapshots toward a diagnostic logic defined by longitudinal and integrative reasoning. The goal is to construct a comprehensive representation of tumour biology by synthesizing heterogeneous data streams: macroscale radiological patterns, microscopic pathological features, and longitudinal clinical trajectories. Decision pathways in this domain are inherently temporal, moving toward adaptive, patient-specific risk modelling where frameworks like DySurv or LILAC attempt to quantify disease evolution and predict immunotherapy outcomes rather than simple malignancy.

There is a clear tension, however, between the theoretical promise of multimodal fusion and its current statistical fragility. While integrating PET/CT deep features with clinical variables can demonstrably improve AUC performance from 0.64 to 0.84, these models are frequently developed on small, paired datasets that foster a high risk of overfitting. A significant concern is that many of these “integrated” architectures are quite brittle; they often fail to maintain internal consistency when faced with the incomplete or irregularly sampled datasets that characterize real-world oncology practice.

The most consequential failure mode in multimodal oncology involves a breakdown in temporal ordering. If an AI highlights a prognostic driver without accounting for the messy reality of clinical records, it risks providing an “illusion of reasoning” that masks a non-generalizable statistical correlation. Achieving true epistemic alignment in this space will require moving beyond simple feature concatenation toward architectures that can signal their own uncertainty when data streams are missing or when macro and micro-level features provide conflicting signals.

Analytically, these domain-specific failures carry divergent translational risks. A failure in breast ultrasound driven by signal noise has an immediate clinical impact, resulting in false-positive biopsies and acute patient anxiety. In contrast, a failure in multimodal oncology stemming from statistical overfitting results in a long-term prognostic risk. Here, the consequence is a breakdown in longitudinal risk stratification, potentially misguiding therapeutic trajectories over months or years rather than impacting a single triage event. Differentiating these ‘consequential failures’ is essential for establishing validation standards that are sensitive to the specific clinical timeline of each discipline.

### 5.3. Human-in-the-Loop Systems in Oncology Imaging

The human-in-the-loop (HITL) paradigm represents a foundational architectural strategy for preserving clinical agencies as autonomous algorithms become embedded in oncology workflows. Rather than positioning the radiologist as a passive recipient of algorithmic outputs, HITL systems refer to the clinician as an active supervisor and collaborative partner in the diagnostic process. This bidirectional “cross-informing” relationship enables continuous refinement of model performance while addressing unresolved concerns regarding authority, accountability, and liability in AI-assisted diagnosis [173].

Unlike static deployment models, HITL frameworks incorporate structured feedback loops that permit real-time auditing of individual predictions and iterative model adaptation. Clinician feedback contributes to high-quality ground truth annotations, facilitates calibration to local imaging protocols, and enhances deployment stability in heterogeneous clinical environments [161].

A major technical challenge in oncologic imaging is the persistent annotation bottleneck; wherein expert labelling of large datasets remains resource intensive. Interactive learning strategies, particularly active learning (AL), mitigate this constraint by selectively querying clinicians for the most informative or uncertain samples. Empirical studies have demonstrated that labelling between 5% and 50% of strategically selected cases can achieve diagnostic performance comparable to that of fully supervised training [174]. AL implementations include pool-based sampling for retrospective uncertainty prioritization, stream-based selective sampling for real-time case triage, and membership query synthesis for generating boundary cases that clarify intergrade distinctions [174].

Effective integration of HITL systems depends on low-friction interfaces embedded within established radiology platforms, such as 3D Slicer or Picture Archiving and Communication System (PACS) environments. Efficient feedback mechanisms including click-based correction, scribble annotation, and gaze-tracking input reduce the interaction burden. The gaze-based bounding box definition for lung nodules, for example, has been shown to significantly decrease physical workload as measured by the NASA Task Load Index [175].

Importantly, the impact of AI support is modulated by clinician experience. Junior practitioners frequently demonstrate improved sensitivity when the sensitivity is augmented by AI assistance, whereas senior radiologists may experience cognitive dissonance when model outputs conflict with established diagnostic intuition. To reconcile these differences, HITL systems increasingly incorporate decision-fusion strategies that weight algorithmic confidence alongside clinician suspicion levels, thereby balancing statistical consistency with experiential expertise.

Within multidisciplinary tumour boards, HITL architectures function as advanced decision-support systems capable of integrating decentralized patient data and streamlining case preparation [170]. Guideline-aware platforms such as PrOPA synthesize patient-specific information with international evidence standards to generate structured recommendations [176]. While emerging large language models demonstrate substantial concordance with expert decisions in standardized scenarios, limitations persist in individualized cases requiring contextual nuance, psychosocial considerations, or complex staging interpretation. These systems remain vulnerable to hallucination and contextual misalignment, particularly in highly specialized domains such as pediatric neuroimaging [177].

The trajectory of clinical AI is therefore moving toward compound architectures that integrate multimodal language models with specialized imaging networks [178]. By bridging the distinction between human-in-the-loop and human-on-the-loop oversight, oncology practice can advance toward collaborative, accountable, and workflow-integrated AI systems that enhance not replace clinical judgment.

### 5.4. Evaluating Explainable AI Beyond Accuracy Metrics

The integration of AI into oncologic imaging exposes a critical tension between technical performance and clinical trustworthiness. While algorithm development traditionally prioritizes predictive accuracy and reproducibility, clinical adoption requires interpretive transparency and alignment with established medical reasoning. A model that achieves high internal validation metrics may nonetheless be clinically rejected if its decision-making logic cannot be meaningfully interpreted.

Although many studies have reported models with strong internal validation metrics, such models may still face barriers to clinical adoption if their decision-making processes are not sufficiently interpretable.

#### 5.4.1. Limitations of Accuracy-Centric Benchmarking

Reliance on overall accuracy as a primary benchmark is increasingly recognized as insufficient in oncology. Internal performance metrics often fail to reflect generalizability across institutions, scanner protocols, or diverse patient populations [179]. In class-imbalanced cancer datasets, accuracy may obscure critical failures in detecting rare but clinically significant malignancies.

Moreover, ground-truth annotations derived from expert labelling or biopsy results are themselves subject to interobserver variability and sampling limitations. Consequently, AI systems that achieve radiologist-level accuracy risk replicating latent biases present in the training data, particularly those stemming from imperfect or subjective ground-truth annotations.

As established in the technical failure modes discussed in Section 3.4.2, shortcut learning further complicates evaluation, as deep learning models may exploit spurious correlations unrelated to pathology (e.g., imaging artifacts or acquisition markers) to achieve high accuracy that does not generalize to real-world clinical settings [180].

Alternative metrics such as the Matthews correlation coefficient (MCC) and the area under the precision–recall curve (AUPRC) provide more informative assessments in class-imbalanced contexts typical of oncology [181]. However, performance metrics alone remain insufficient without parallel evaluation of interpretability and reliability.

#### 5.4.2. Technical Evaluation of Explainability

Explainable AI methods are broadly categorized into intrinsic models, which are interpretable by design, and post hoc techniques applied to opaque architecture. Their evaluation generally focuses on three core criteria: faithfulness, stability, and human-centred utility.

As established in Section 3.4.3, fidelity [182] remains a core criterion for XAI evaluation. Beyond its conceptual definition, algorithmic validation of fidelity requires rigorous strategies such as feature perturbation and deletion–insertion testing. Comparative studies indicate variability in fidelity across explanation methods, with performance dependent on architecture, imaging modality, and task complexity.

Stability represents another critical dimension. Post hoc explanations may exhibit high sensitivity to minor, nondiagnostic perturbations. Noise analyses have shown substantial degradation in SHAP stability under small input variations in ophthalmologic imaging tasks. Robustness is typically quantified using L_2_ or L∞ perturbation norms [183]. Without stability guarantees, fluctuating heatmaps may undermine clinician confidence.

### 5.5. Clinical Validation and Human–AI Interactions

Beyond algorithmic metrics, explainability must demonstrate measurable benefit within real clinical workflows. Radiologist-in-the-loop studies have indicated that AI-augmented reading can increase cancer detection rates and reduce workload. However, these gains are tempered by the risk of automation bias, wherein clinicians may over rely on AI recommendations and demonstrate reduced independent diagnostic vigilance when assistance is withdrawn [184].

The impact of AI assistance is strongly experience dependent [185]. Multireader multicase analyses consistently revealed that junior radiologists achieved greater sensitivity improvements, whereas senior experts may perceive marginal incremental value. This divergence is illustrated by challenging benchmarks such as Radiology’s Last Exam (RadLE), where expert radiologists achieved 83% accuracy compared with approximately 30% for frontier generalist AI models [186].

Large-scale prospective trials such as MASAI [187] and NELSON [188] provide emerging evidence that AI can reduce radiologist workload by up to 44% in certain screening contexts while maintaining noninferior detection rates and identifying patient subgroups with improved survival outcomes. These studies represent critical steps toward bridging the deployment gap between retrospective validation and real-world integration.

### 5.6. Uncertainty Estimation and Trust Calibration

Clinical trust in oncology AI requires explicit quantification of predictive uncertainty. Bayesian deep learning frameworks distinguish between aleatoric uncertainty, arising from inherent data noise (e.g., motion artifacts and partial volume effects), and epistemic uncertainty, reflecting model ignorance due to limited or nonrepresentative training data [69].

Bayesian deep learning (BDL) formalizes uncertainty by modelling network weights as probability distributions rather than fixed parameters [189]. The predictive posterior for a new instance is obtained by marginalizing over the parameter distributions:

(5) $$p\left(y|x^{*},X,Y\right)=\int p\left(y|x^{*},\omega \right)\;p\left(\omega |X,Y\right)\;d\omega$$

Although theoretically robust, exact inference remains computationally intractable for high-dimensional networks, necessitating approximation strategies such as variational inference, deep ensembles, or Markov chain Monte Carlo sampling [190].

Monte Carlo dropout provides a computationally efficient approximation by performing multiple stochastic forward passes at test time [191,192,193]. In digital histopathology, uncertainty thresholding has achieved near-perfect classification accuracy after ambiguous cases have been rejected. However, compared with deep ensembles, MC dropout may yield less well-calibrated estimates and may inadequately separate epistemic uncertainty from aleatoric uncertainty.

Emerging single-network methods such as evidential deep learning directly parameterize Dirichlet distributions to capture higher-order uncertainty and detect out-of-distribution samples [194]. Training-time strategies, including online label smoothing, further improve calibration while preserving discrimination between malignant and benign tissue [195].

Calibration the alignment between predicted probabilities and observed outcomes is central to clinical deployment [196]. Metrics such as expected calibration error (ECE), expected segmentation calibration error (ESCE), and harmonic Dice (HDice) extend reliability assessment to segmentation tasks [197]. Post hoc techniques, including temperature scaling, isotonic regression, and parameterized temperature scaling, reduce miscalibration and improve probability interpretability [80,198,199].

In federated learning contexts, uncertainty estimates help identify domain shifts and site-specific overconfidence. Systems such as PICTURE leverage uncertainty thresholding to prevent silent failure when rare tumour subtypes are encountered [200]. Nonetheless, a trade-off may arise between fairness interventions and uncertainty reliability, as bias mitigation strategies can inadvertently degrade calibration quality [201].

Embedding uncertainty estimation within HITL workflows transforms probabilistic modelling into actionable triage strategies. Radiologists demonstrate greater acceptance of AI recommendations when confidence estimates are transparent and well calibrated, reducing override rates [202]. Spatial visualization approaches such as uncertainty-CAM further localize regions of model doubt. The emerging field of explainable uncertainty estimation (XUE) seeks to unify interpretability and probabilistic modelling by clarifying not only what the model predicts but also why it is uncertain when distinguishing between data limitations and model knowledge gaps.

Collectively, human-in-the-loop architectures, rigorous explainability evaluation, human–AI performance studies, and uncertainty-aware calibration frameworks constitute the technical and operational foundations of trust in oncology imaging AI. However, calibrated interaction at the model and workflow level does not resolve broader challenges related to regulatory governance, ethical accountability, lifecycle monitoring, and system-level oversight. The translation of trustworthy AI into routine oncologic practice therefore requires alignment not only between clinicians and algorithms but also between technological capability and institutional, legal, and societal frameworks. These broader dimensions are addressed in the subsequent section.

### 5.7. Case Studies of Prospective Validation and Clinical Challenges

This section stratifies evidence by validation maturity, highlighting the transition from Tier 1 retrospective benchmarks to the Tier 3 real-world impact observed in landmark prospective trials. This distinction is vital, as many XAI limitations identified in Tier 1 studies persist or amplify during clinical deployment. What is perhaps more concerning is that these issues persist even in studies reporting strong predictive performance, raising questions about how reliably such systems can be translated into practice.

One of the more immediate concerns relates to the stability of explanations themselves. In prostate MRI, a convolutional neural network trained on the ProstateX dataset combining T2-weighted images with basic clinical variables was able to localize lesions with a relatively small error (6.93 pixels) [203]. However, when different post hoc explanation methods were applied, including Grad-CAM, LIME, SHAP, and saliency maps, the resulting heatmaps did not agree with one another. Each method appeared to explain the same prediction differently. While all of them produced outputs that looked reasonable at first glance, the lack of consistency makes it difficult to interpret what the model is using. A similar issue appears in lung nodule classification using the LIDC-IDRI dataset, where models trained on different data splits produced comparable predictions, but their explanation maps showed almost no structural similarity [204]. In that case, the instability was not across methods, but across training runs. Taken together, these findings suggest that explanation variability raises a more fundamental concern about whether these explanations can be trusted as representations of model reasoning.

A different set of issues becomes visible when looking at what the model is learning. In digital pathology, a GoogLeNet model trained on TCGA cohorts (melanoma, breast, and lung cancer) was analysed using Layer-wise Relevance Propagation [205]. The resulting maps indicated that the model was often relying on staining artefacts and dataset-specific patterns rather than tumour morphology. What makes this case particularly convincing is that removing these biases improved performance, suggesting that the model was not just using irrelevant features, but was being actively misled by them.

#### 5.7.1. Technical Fragility: Instability, Fidelity, and Computational Constraints

A recurring concern across several studies is that explanation methods themselves introduce an additional layer of variability, which is not always apparent when looking at predictive performance alone. In neuro-imaging applications, for example, convolutional neural networks including ResNet50, VGG16, and Inception-v3 achieved comparable classification accuracy, yet the corresponding LIME explanations differed substantially for the same input images. Despite similar model outputs, the highlighted regions were not consistent across architectures, suggesting that explanation behaviour may depend as much on model structure as on the underlying data. An additional observation in this study was that clinicians tended to interpret these explanations as confirmatory evidence, even when the underlying attribution was unstable, pointing to a subtle risk of confirmation bias.

A similar pattern emerges in brain tumour classification tasks, where multiple gradient-based explanation methods such as Grad-CAM++, EigenGradCAM, and Ablation-CAM were evaluated on the same model outputs. While all methods produced visually plausible saliency maps, they frequently disagreed on the regions of importance. This inconsistency was compounded by practical constraints: some methods required up to 7.95 s per image to generate explanations, raising questions about their feasibility in time-sensitive clinical settings. Here, the issue is not only disagreement between methods, but also the computational overhead associated with generating explanations, which may limit their routine use.

In digital histopathology, the problem takes a slightly different form. A controlled experiment comparing Automated Concept-based Explanations (ACE) with Grad-CAM demonstrated that the two approaches could attribute model predictions to entirely different features, even when the model itself was fixed. Artificially introduced features such as coloured patches embedded in the image were identified differently depending on the explanation technique. While ACE tended to isolate concept-level features, Grad-CAM produced broader, less specific activation patterns. This divergence highlights a tension between methods that aim for semantic interpretability and those that provide spatial localization, with no clear indication of which better reflects the underlying decision process.

These individual observations are reinforced by broader evidence. A meta-analysis spanning 67 studies reported that explanation methods failed to accurately represent model reasoning in approximately 55–68% of cases [9]. This finding suggests that the issue is not confined to specific datasets or architectures but may reflect a more general limitation of current XAI approaches. Notably, the analysis included a range of modalities and explanation techniques, indicating that instability and limited fidelity are not restricted to a particular class of methods.

#### 5.7.2. Clinical–Epistemic Misalignment: Lack of Alignment with Clinical Reasoning

Beyond technical instability, a more subtle but equally important limitation lies in the disconnect between what models learn and how clinicians’ reason about disease. Several studies suggest that even when explanations are available, they do not necessarily correspond to clinically meaningful features or decision pathways.

In multimodal settings, this issue becomes particularly visible. In one study evaluating vision–language models for medical reasoning, systems capable of generating detailed explanatory text achieved only around 30% diagnostic accuracy, compared to approximately 83% for radiologists [186]. What is notable is not just the performance gap, but the nature of the explanations: the models produced coherent, step-by-step reasoning traces that appeared plausible on inspection, yet were frequently incorrect. The visual and textual components were often loosely coupled, with correct identification of image features not translating into correct clinical conclusions. This suggests that explanation generation can operate independently of true diagnostic reasoning, creating an illusion of understanding.

A related issue is observed in prototype-based architectures designed explicitly to improve interpretability. In ProtoCaps-style models, predictions are justified through similarity to learned prototypes, which are intended to represent meaningful clinical patterns [71]. However, closer analysis shows that the selected prototypes do not always correspond to the features driving the model’s decision. In some cases, prototypes associated with one class were used to justify predictions for another, indicating a disconnect between the explanation layer and the underlying feature space. While the model remains accurate at a classification level, the reasoning it presents is not reliably aligned with its internal computations.

Even in more conventional imaging tasks, similar concerns arise. In neuro-imaging studies using LIME, clinicians were observed to interpret highlighted regions as causal indicators of disease, despite the explanations being based on local perturbations and correlations rather than causal mechanisms. This misinterpretation is not simply a user error; it reflects a broader mismatch between how explanations are generated and how they are understood in clinical contexts. When explanation outputs resemble familiar diagnostic cues, they may be over-interpreted, even if their underlying basis is weak.

#### 5.7.3. Deployment Challenges: Human–AI Interaction and Cognitive Effects

When these systems are evaluated closer to real clinical workflows, a different set of limitations becomes apparent less about model behaviour in isolation and more about how explanations are interpreted and used by clinicians. In this setting, the effectiveness of XAI depends not only on technical fidelity but also on usability, timing, and cognitive integration.

Evidence from screening workflows illustrates this tension. In a large-scale breast cancer screening setting, AI-assisted triage was shown to reduce radiologist workload by approximately 44%, primarily by filtering out low-risk cases. While this represents a clear operational advantage, the study also highlights unresolved concerns regarding validation endpoints and the potential for automation bias. In particular, the reliance on AI outputs in high-throughput settings raises the possibility that errors may go unchecked, especially when explanations are not systematically reviewed or are perceived as secondary to the prediction itself.

More controlled usability studies point to similar challenges at the individual level. In breast cancer clinical decision support systems, clinicians were asked to interpret model outputs alongside explanation visualizations under realistic time constraints. Although explanations were intended to support decision-making, participants often reported difficulty interpreting them, particularly when multiple features or regions were highlighted simultaneously. In some cases, explanations were considered too abstract to be actionable, while in others they increased cognitive load without improving confidence in the decision. These findings suggest that the design and presentation of explanations are critical, and that interpretability does not automatically translate into usability.

There is also evidence that clinicians engage with AI outputs selectively, depending on context. Observational studies of radiologist workflow indicate that AI tools are more frequently consulted in cases that appear straightforward, such as normal or low-risk images, while more complex cases are handled independently. This selective usage introduces an additional layer of bias, as the system’s influence is not uniform across case types. In such scenarios, explanations may reinforce existing tendencies rather than provide meaningful support where it is most needed.

#### 5.7.4. Validation Crisis: Generalization, Domain Shift, and Dataset Bias

Questions of generalizability remain one of the more persistent challenges, and the case studies considered here suggest that explainability does little to mitigate this issue on its own. In digital pathology, for instance, models trained and validated on a single dataset often show a noticeable drop in performance when applied to external cohorts such as CPTAC or Mayo Clinic data [84]. What is particularly concerning is that these models tend to remain highly confident in their predictions even when they are wrong, with out-of-distribution cases going undetected in a substantial proportion of instances. Explanation methods, when applied in this context, frequently highlight features that are consistent within the training dataset but do not generalize, making it difficult to distinguish between true pathology and dataset-specific artefacts.

A similar pattern is observed in multicentre prostate MRI studies, where models trained across institutions show improvements in sensitivity, yet still struggle to maintain consistent performance across scanners and acquisition protocols. The variability introduced by differences in imaging hardware, reconstruction settings, and patient populations appears to affect both prediction and explanation. In such cases, explanations may reflect scanner-specific characteristics rather than underlying disease features, which limits their clinical interpretability.

The issue becomes more pronounced in distributed or federated settings. Studies examining models trained across multiple hospitals with non-identically distributed data report substantial drops in performance on the order of 40% in F1 score when models are transferred between sites [55]. These findings suggest that model behaviour is tightly coupled to local data distributions, and that aggregation alone does not resolve underlying heterogeneity. From an interpretability perspective, this raises an additional concern: explanations generated in one setting may not be meaningful, or even valid, in another.

#### 5.7.5. Hybrid Failure Modes: Interacting Limitations and Compounded Effects

While the preceding categories highlight distinct limitations, several case studies suggest that these issues rarely occur in isolation. Instead, they tend to interact, giving rise to more complex failure modes in which instability, misalignment, and lack of generalizability reinforce one another.

Concept bottleneck models provide one such example. These architectures are explicitly designed to improve interpretability by structuring predictions around intermediate, human-interpretable concepts. In practice, however, they remain sensitive to shifts in the underlying data distribution, with observed degradation in both predictive performance and conceptual consistency across settings. Even when concept-level explanations are available, they do not always remain stable and may drift in ways that are difficult to detect without additional validation. This suggests that introducing an interpretable intermediate layer does not fully resolve the underlying issues of robustness and generalization.

A related pattern is observed in prototype-based approaches. In models such as ProtoCaps, predictions are justified through similarity to learned prototypes, which are intended to represent clinically meaningful patterns [71]. However, as noted earlier, these prototypes do not always correspond to the features driving the prediction. When combined with dataset biases or shortcut learning, this can lead to situations where the model achieves high accuracy while relying on incorrect or non-generalizable reasoning pathways. In such cases, the explanation mechanism does not fail independently; rather, it reflects and amplifies underlying weaknesses in the model.

Bias-sensitive analyses in histopathology further illustrate how these effects can compound. In studies comparing different explanation methods, models were shown to rely on artificial features such as colour artefacts while also producing explanations that varied depending on the method used. Here, instability and bias are intertwined: the model learns spurious features, and the explanation methods provide inconsistent accounts of that learning. This makes it particularly difficult to diagnose the problem, as neither the prediction nor the explanation alone provides a complete picture.

More broadly, these hybrid cases highlight a practical concern. When multiple limitations are present simultaneously, addressing any single issue whether through improved explainability, better data curation, or architectural changes may not be sufficient. Instead, the interaction between factors becomes the dominant challenge. Explanations may appear coherent, predictions may remain accurate, and yet the system may still behave in ways that are difficult to anticipate or justify.

Across these prospective scenarios, recurring patterns emerge namely instability of explanations, increased cognitive burden, and a persistent mismatch between algorithmic outputs and clinical reasoning. These observations are not isolated failures but manifestations of deeper structural limitations in current XAI approaches, which we systematically categorize in the following section

### 5.8. Critical Limitations of XAI in Clinical Practice

Drawing on the cumulative evidence presented throughout this review including prior methodological work, empirical studies, and the case-based analyses in Section 5.7 it becomes clear that the limitations of explainable artificial intelligence (XAI) in clinical oncology are systemic rather than incidental. Despite advances in interpretability techniques, current XAI approaches remain fundamentally misaligned with the technical, cognitive, and epistemic requirements of real-world clinical practice.

These limitations can be organized into four principal domains: technical fragility, deployment-related cognitive burden, clinical–epistemic misalignment, and a broader validation crisis. Collectively, they define the core translational barriers that impede the effective clinical adoption of XAI.

#### 5.8.1. Technical Fragility

The stability and fidelity gap highlights fundamental weaknesses in current explainability methods, particularly in terms of robustness, consistency, and faithfulness to underlying model behaviour. This gap manifests through the following issues:

- Sensitivity to Noise: The exploratory transparency layer is characterised by a significant ‘stability and fidelity gap’, where post hoc maps are highly sensitive to noise.
- The Disagreement Problem: Because the explanatory layer often produces conflicting rationales for the same image (the ‘disagreement problem’), it cannot yet be viewed as a ‘ground truth’ for medical conclusion-making.
- Fidelity vs. Plausibility: False confidence where an explanation appears clinically reasonable but does not actually reflect the model’s true global decision logic.

#### 5.8.2. Deployment Challenges

The deployment of explainability methods introduces significant human-factor challenges, particularly in the form of increased cognitive burden and altered decision behavior. This “cognitive load exploitation” is evident through the following effects:

- Increased Workload: Instead of streamlining workflows, AI-generated heatmaps can increase reporting time by 33% and delay dictation by over 40%. This cognitive load exploitation occurs because interpreting the explanation becomes more demanding than the original diagnostic task.
- Authority Modulation: The liability-driven behavior where clinicians feel compelled to scrutinize every AI-highlighted region to mitigate perceived risk, leads to fatigue.
- Automation Bias: This is the ethical risk where clinicians may over-rely on AI recommendations, leading to a loss of independent diagnostic vigilance.

#### 5.8.3. Clinical–Epistemic Misalignment

A further limitation arises from a fundamental disconnect between computational representations and clinical reasoning. This “clinical–epistemic misalignment” reflects the gap between pixel-level pattern recognition and the concept-level understanding required in medical practice. The implications of this misalignment are evident in the following:

- Lack of Contextual Reasoning: Models often fail to replicate the contextual reasoning used by experts, such as incorporating longitudinal patient history or prior biopsy results. For example, in prostate cancer, AI often lacks the ability to resolve equivocal lesions using the multi-parametric data points (like PSA levels) that radiologists routinely use.
- Shortcut Learning: Saliency maps often highlight spurious correlations (scanner artifacts, skin markings, or pen annotations) rather than true pathology. This “illusion of interpretability” can lead a clinician to trust a model that is “right for the wrong reasons”.

#### 5.8.4. Validation Crisis

The validation crisis highlights a fundamental limitation in current explainability research: the reliance on technical evaluation metrics in the absence of robust clinical validation. This gap is evident in the following aspects:

- Technical Proxies vs. Clinical Utility: Most XAI research relies on technical metrics (faithfulness, sparsity) rather than prospective clinical trials that measure improved patient outcomes or safer therapeutic choices.
- The Interpretability–Performance Paradox: The most accurate models (e.g., Vision Transformers) are often the least transparent, creating a barrier for high-stakes oncology.

These limitations emphasize that strong Tier 1 performance does not equate to clinical readiness. Current XAI approaches remain fundamentally distanced from the Tier 3 validation required for routine oncology, as they highlight where a model looks but fail to explain why a medical conclusion was reached within a specific clinical context. Importantly, these challenges are not independent but often interact, reinforcing one another and amplifying translational risk. Addressing them therefore requires not incremental methodological improvements, but a more coordinated shift in how explainability is designed, evaluated, and integrated into clinical systems.

### 5.9. Research Priorities and Future Directions

A priority is the systematic evaluation of explanation fidelity and stability. Current approaches rely heavily on qualitative inspection, which is insufficient for clinical use. There is a need for standardized, quantitative benchmarks that assess consistency across explanation methods, model initializations, and input perturbations.

Second, improving alignment between model reasoning and clinical knowledge remains essential. This will likely require integrating domain-informed constraints into model design, ensuring that explanations correspond to clinically verifiable features rather than purely statistical patterns.

Third, human–AI interaction must be explicitly addressed. Rather than treating explanations as static visual outputs, future work should evaluate how clinicians interpret and use these systems in practice, including their impact on cognitive load, trust calibration, and decision-making behaviour.

A fourth priority is robust external validation across heterogeneous datasets and institutions. Explainability methods may help reveal biases but cannot replace rigorous evaluation under real-world variability.

Fifth, accountability requires more than feature attribution. There is a need for explanation frameworks that provide structured, evidence-based reasoning pathways that clinicians can interrogate and justify.

Finally, future work should consider compound failure modes, where instability, bias, and misalignment interact. Addressing these challenges will require integrated evaluation frameworks that move beyond isolated performance metrics.

## 6. Bridging the Trust Gap

This section discusses the technical fragility and human-centric friction that occur during clinical deployment.

### 6.1. Regulatory Evolution and Lifecycle Management

Regulatory oversight of artificial intelligence (AI) in oncology is transitioning from a static approval paradigm toward a dynamic, lifecycle-based governance model that acknowledges the potential for performance drift, dataset shift, and iterative model adaptation. This evolution reflects fundamental recognition: unlike conventional medical devices, AI systems are probabilistic and data dependent and require continuous evaluation beyond initial market authorization.

In the U.S., the U.S. Food and Drug Administration (FDA) has operationalized this perspective through the total product life cycle (TPLC) framework for AI-enabled device software functions (AI-DSF). The TPLC model extends regulatory scrutiny across design, validation, deployment, and post market monitoring. A central innovation within this approach is the Predetermined Change Control Plan (PCCP), which enables manufacturers to prospectively define permissible modifications such as retraining on demographically diverse datasets or updating validation protocols within an approved regulatory envelope. By allowing structured, preauthorized updates without requiring new premarket approval (PMA) submissions, the PCCP mechanism balances innovation with accountability if developers maintain transparent documentation, rigorous performance monitoring, and predefined safety thresholds.

In contrast, the European Union has implemented the EU AI Act, a sector-agnostic regulatory framework that classifies most oncology-oriented AI systems as “high risk.” This designation imposes stringent obligations, including comprehensive risk management systems, high-quality and representative training datasets, traceability requirements, and enforceable human oversight mechanisms that permit clinicians to intervene in or override automated outputs. While the AI Act strongly emphasizes transparency, bias mitigation, and fundamental rights protection, it does not yet provide an update pathway equivalent to the FDA’s PCCP. Consequently, developers may face regulatory inertia, whereby models remain technically static to preserve conformity, potentially limiting adaptive improvement.

These regional frameworks are increasingly shaped by international standardization efforts, including International Organization for Standardization technical specifications such as ISO/IEC TS 6254 [206] and ISO/IEC TR 24028 [207], which articulate trustworthiness dimensions, including reliability, robustness, fairness, accountability, and transparency. Together, these regulatory trajectories signal a global shift toward lifecycle governance models in which explainability, monitoring, and documentation are not optional enhancements but structural prerequisites for clinical translation.

### 6.2. Ethical and Legal Considerations

Beyond regulatory compliance, the integration of AI into oncological workflows raises complex ethical and medico-legal challenges that directly influence trust calibration. Algorithmic bias remains one of the most consequential risks. Bias may arise from imbalanced training data, limited demographic representation, or cross-institutional heterogeneity, leading to measurable reductions in diagnostic sensitivity among minority populations or patients with specific physiological characteristics, such as high breast density. Such disparities undermine both clinical validity and ethical legitimacy.

Although mitigation strategies including adversarial debiasing, dataset reweighting, and external multicohort validation have demonstrated promise, structural limitations persist. Notably, only a small proportion of publicly available datasets report comprehensive demographic metadata, constraining meaningful bias auditing and subgroup performance analysis. Without transparent reporting of demographic composition and stratified performance metrics, claims of fairness remain empirically underdetermined.

A further concern is automation bias, wherein clinicians may disproportionately rely on AI output despite contradictory clinical evidence. This cognitive tendency is particularly pronounced among less experienced practitioners and may distort diagnostic reasoning pathways. As discussed in Section 5, this underscores the necessity of calibrated explainability systems must support, rather than supplant, clinical reasoning.

The legal landscape is simultaneously evolving. Traditional liability doctrines, such as the Learned Intermediary Doctrine, position the physician as the principal decision maker responsible for weighing risks and benefits. However, when AI systems operate as opaque intermediaries when manufacturers cannot fully explicate algorithmic logic and clinicians cannot independently verify outputs the integrity of this communicative chain becomes strained.

The European Union’s New Product Liability Directive (PLD) 2024 further recalibrates accountability by introducing strict (no-fault) liability standards for defective AI systems, potentially expanding developer responsibility in cases of harm. In this environment, adherence to structured reporting frameworks such as CONSORT-AI and SPIRIT-AI becomes essential. These guidelines mandate transparent disclosure of AI versioning, training data characteristics, human–AI interaction dynamics, error analysis, and performance variability, thereby operationalizing accountability in clinical research and deployment contexts.

Ultimately, bridging the trust gap in oncology AI requires convergence across three interdependent pillars: adaptive regulatory oversight, empirically validated and semantically meaningful explainability methods, and a robust ethical–legal infrastructure grounded in equity, accountability, and patient safety. AI systems can transition from technically impressive tools to clinically trusted partners in oncologic decision-making only through this multidimensional alignment.

### 6.3. Integrative Synthesis

The central challenge identified in this review is not whether AI can achieve high predictive accuracy, but whether its reasoning can be integrated into clinical practice in a manner that sustains professional accountability. By synthesizing the evidence across our five core research objectives, we can move beyond isolated technical benchmarks toward a comprehensive understanding of how explainability serves as the link between algorithmic output and oncological decision-making.

#### 6.3.1. Addressing Key Challenges (RQ1)

Our investigation into the primary obstacles to adoption suggests that the “trust gap” is far more complex than simple model opacity. A fundamental concern emerging from the literature is the prevalence of dataset bias and shift, which often results in brittle representations that fail when moved between institutions. This is best illustrated by the “prostate cancer generalization crisis,” where models that initially appeared accurate saw their diagnostic performance plummet when faced with hardware from different scanner vendors. We also find that models frequently succumb to shortcut learning, mistakenly grounding predictions in non-biological artifacts like scanner noise or skin markings rather than true pathology. When these technical flaws are compounded by explanation instability and a fragmented regulatory landscape, it becomes clear that current deep learning systems face structural hurdles that no amount of post hoc visualization can fully resolve.

#### 6.3.2. Impact of XAI on Clinician Trust (RQ2)

The investigation into how explainable AI (XAI) methods influence the professional user experience (RQ2) suggests that trust in oncological deep learning is best understood as a calibrated, relational process that develops between the clinician and the system. While our synthesis of the literature indicates that explainable tools can measurably bolster clinician confidence and reduce false-positive rates, this effect remains inherently fragile. We found that durable trust is rarely built on high performance metrics alone; instead, it requires that model explanations are faithful to the underlying logic, semantically meaningful to the specialist, and uncertainty aware.

A recurring observation across the reviewed studies is the precarious distinction between perceived trust and justified trust. Persuasive but uncalibrated visualizations, such as diffuse saliency maps, can inadvertently mask model failures and induce overconfidence or automation bias. The evidence regarding RQ2 confirms that when an AI system provides an incorrect recommendation or a misleading visual rationale, it often leads to a rapid erosion of confidence. This underscores a critical finding: a model’s ability to communicate its own epistemic limits through uncertainty quantification is ultimately as vital to the clinical partnership as the prediction itself.

Finally, the synthesis highlights that the practical utility of XAI is often limited by sociotechnical hurdles like “cognitive load exploitation”. Instead of streamlining the diagnostic process, poorly integrated visual overlays can increase reporting times by approximately 33%, as clinicians may feel a secondary pressure termed “authority modulation” to exhaustively scrutinize every AI-flagged region due to perceived liability risks. Consequently, the findings regarding RQ2 suggest that the transition from a “black box” to a trusted clinical partner depends less on visually persuasive heatmaps and more on the system’s ability to provide reliable, verifiable performance boundaries.

#### 6.3.3. Techniques for Improving Interpretability (RQ3)

The synthesis of technical modifications identifies a significant industry pivot from retrospective fixes toward a philosophy of “transparency by design”. We find that concept-bottleneck models and prototype-based reasoning offer a promising path forward because they restructure the neural pipeline to mirror human logic. By decomposing a diagnosis into recognizable clinical descriptors such as mass margins or cellular density these systems allow oncologists to verify the model’s reasoning in real-time. Additionally, the literature points to the success of attention regularization and causality-aware architectures in suppressing the background noise that often distracts traditional deep learning models. These design-level changes provide a much more stable foundation for trust than the often-erratic approximations of post hoc tools.

#### 6.3.4. Alignment with Clinical Reasoning (RQ4)

A fundamental realization from this study is that technical transparency, in isolation, does not guarantee clinical utility. Epistemic alignment should not be treated as a universal or static performance metric; rather, it is a relational property that must be redefined for each discipline’s unique “semantic anchoring”. Achieving this alignment requires a shift where model outputs are no longer expressed as abstract, pixel-level heatmaps but are instead translated into recognized diagnostic constructs such as BI-RADS, PI-RADS, or TNM staging descriptors.

For instance, in breast imaging, grounding predictions in familiar morphological features such as spiculated margins or calcification patterns allows for the verification of pathophysiologically sound evidence. Conversely, in multimodal oncology, the logic must anchor to TNM descriptors or nodal involvement to be relevant for multidisciplinary staging. The current tendency to celebrate high AUC scores while ignoring these semantic foundations is a significant oversight; a model that lacks this anchoring remains “clinically misaligned” regardless of its statistical accuracy.

Furthermore, true alignment depends on the system’s ability to handle diagnostic complexity, such as the contextual synthesis of longitudinal patient history, prior biopsies, or PSA levels used to resolve ambiguous cases like PI-RADS 3 lesions. Ultimately, when AI functions as a “logic-concordant verification layer” rather than an “opaque authority”, it can successfully mirror the multidisciplinary “tumour board” approach, facilitating a more accountable and defensible form of decision support. This strategic shift from exploratory visual approximations toward domain-specific epistemic alignment is the only viable pathway for transforming experimental prototypes into dependable clinical partners.

#### 6.3.5. Impact on Diagnostic Performance and Confidence (RQ5)

The final dimension of this synthesis evaluates whether explainability translates into tangible gains in diagnostic performance. The evidence suggests that the clinical utility of these systems is most pronounced when they function as part of a collaborative human–AI synergy rather than as autonomous predictors. In these settings, explainable tools frequently surpass both unassisted clinicians and standalone opaque models, yielding measurable improvements in sensitivity and a reduction in missed lesions. These gains are driven by a model’s ability to expose its internal logic, allowing specialists to detect failures such as shortcut learning or institutional bias and intervene through human-led refinement.

However, the impact on clinician confidence is nuanced. While explanations consistently boost a user’s feeling of security, this trust is inherently fragile if the visual rationale is uncalibrated or misleading. Because persuasive but incorrect visualizations can mask model fragility and induce overconfidence, calibration becomes the central requirement for clinical safety. By using uncertainty quantification and selective prediction, models can communicate their epistemic limits and defer high-ambiguity cases to an expert. In high-stakes oncology, the ability to signal when a model is “unsure” is arguably as vital as the prediction itself, as trustworthiness depends less on visual appeal and more on the reliability of performance boundaries.

Table 7 illustrates this distinction by contrasting “visually persuasive” methods, which may be grounded in artifacts, with “trust calibrating” approaches that promote epistemic alignment with clinical reasoning.

#### 6.3.6. A Framework for Evidence Maturity and Readiness

When viewed collectively, the evidence synthesized in this review suggests a need to reposition explainability from a supplementary visual aid to a structural design principle. To bridge the persistent “translational gap,” we define the following hierarchy of evidence maturity:

Tier 1: Proof-of-Concept. Characterised by retrospective, single-centre validation. These studies demonstrate technical feasibility but lack evidence of real-world stability.

Tier 2: External Validation. Involves multicentre, multi-vendor datasets. This tier addresses the ‘generalization crisis’ and site-transfer degradation observed in 80% of radiological models.

Tier 3: Prospective Clinical Evidence. Represented by large-scale trials (e.g., MASAI, NELSON). This is the highest evidentiary weight, confirming that AI-assisted workflows improve patient survival and reduce clinician workload in routine practice.

Tier 1 (Proof-of-Concept) primarily represents an exploratory phase of development, where post hoc XAI methods such as Grad-CAM or LIME provide initial computational transparency within controlled, retrospective environments. While these exploratory methods may bolster “perceived trust” by providing visually intuitive highlights, they lack the “justified trust” required for integration into mature clinical infrastructure, as they often fail to accurately reflect the model’s true internal decision logic.

Consequently, the strategic pathway to transforming a Tier 1 prototype into a Tier 3 dependable clinical partner requires moving beyond these exploratory approximations toward “epistemic alignment”. This transition ensures that the AI system functions not as an opaque authority but as a logic-concordant verification layer whose reasoning is structurally harmonized with the hierarchical and contextual diagnostic processes used in routine oncology.

This investigation reveals a distinct hierarchy of readiness across our research objectives. In the context of systematic barriers (RQ1), we find that obstacles like shortcut learning are often latent in Tier 1 benchmarks but manifest as catastrophic failures during Tier 2 validation. Similarly, regarding clinician trust and diagnostic performance (RQ2 and RQ5), while Tier 1 results show that saliency maps increase perceived trust, Tier 3 evidence remains essential to ensure that this trust is justified and leads to improved collaborative decision-making.

Technical analysis of architectural solutions (RQ3) establishes that intrinsic designs, such as concept-bottleneck and prototype networks, offer superior transparency over post hoc tools by mitigating the “illusion of interpretability.” Ultimately, meaningful clinical integration depends on the epistemic alignment explored in RQ4 a critical prerequisite for transforming a Tier 1 prototype into a Tier 3 dependable clinical partner. By adopting this translational hierarchy, it becomes clear that the path to routine oncology practice requires the transformation of experimental prototypes into accountable and clinically dependable infrastructure.

#### 6.3.7. A Progressive Model of Trust Integration

Synthesizing the outcomes of our five core research objectives suggests that the path to clinical adoption is a three-stage developmental journey. However, this stage remains limited by the inherent instability and fidelity gaps common in post hoc approximations, which can lead to misinterpretations of model behaviour and reduced trust in the model’s outputs.

The second phase moves toward clinical–epistemic alignment. Here, the focus shifts from simply showing “where” a model looks to structuring explanations that mirror the actual diagnostic reasoning frameworks used in oncology. This alignment is a necessary bridge, but true integration is only achieved in the third stage: collaborative calibration and performance integration. In this phase, explanations are no longer isolated features but are embedded within uncertainty-aware, human-in-the-loop systems that demonstrably enhance diagnostic outcomes.

The trust gap remains wide as long as AI systems are confined to technical transparency alone. While semantic alignment with medical reasoning begins to narrow this divide, the gap truly closes only when that alignment translates into measurable performance gains within a demonstrably accountable clinical ecosystem.

These findings collectively suggest that explainability must be repositioned from a simple visualization tool to a core structural design principle. In this expanded role, it directly informs diagnostic accuracy, the mitigation of bias, and the overall legal defensibility of the system. Given that diagnostic errors in oncology carry such profound consequences, it is no longer sufficient for AI to be merely accurate; it must also be epistemically aligned with clinical reasoning, aware of its own uncertainty, and deeply embedded within institutional workflows.

Moving forward, research should shift away from isolated methodological experiments in favor of integrated strategies that unify semantic alignment with prospective validation and regulatory oversight. The following Section 6.4 builds upon this synthesis to outline specific strategic directions for transforming explainable AI from a promising area of research into a dependable component of clinical infrastructure.

### 6.4. Strategic Directions

Bridging the trust gap in oncology AI requires coordinated advancement across methodological design, clinical validation, regulatory integration, and sociotechnical governance. Building upon the synthesis of RQ1–RQ5, future progress should prioritize five interdependent strategic directions.

#### 6.4.1. Embedding Semantic Alignment into Model Architecture

Future systems should move beyond post hoc visualization toward architectures that encode clinically meaningful abstractions during training. Concept-bottleneck networks, prototype-based learning, structured attention constraints, and multimodal fusion frameworks must be systematically evaluated not only for interpretability but also for epistemic alignment with diagnostic reasoning. Research should explicitly test whether hierarchical feature representations correspond to established oncologic taxonomies and reporting lexicons. Embedding semantic structure at the architectural level reduces reliance on retrospective explanations and promotes traceable reasoning pathways that are auditable across model updates.

#### 6.4.2. Standardizing Trust and Calibration Metrics

Current interpretability metrics (e.g., saliency overlap or concept accuracy) remain insufficient proxies for clinical trust. Prospective studies should incorporate calibration error, uncertainty communication quality, diagnostic confidence alignment, override behavior, and workflow impact measures. Trust should be quantified longitudinally, including sensitivity to incorrect AI outputs and recovery dynamics following model errors. Standardized reporting of human–AI interaction metrics, analogous to performance benchmarks, would enable meaningful cross-study comparison and reduce reliance on subjective trust claims.

#### 6.4.3. Advancing Prospective and Multicentre Validation

Most explainability evaluations remain retrospective and dataset bound. Future research must prioritize prospective, multicentre clinical studies assessing real-world deployment, performance drift, and demographic robustness. Explainability mechanisms should be evaluated under domain shift conditions to determine whether they support early detection of bias or degradation. Integration with structured reporting systems and multidisciplinary tumour boards should be explicitly examined to assess whether explanations enhance collaborative decision-making rather than merely individual interpretation.

#### 6.4.4. Designing for Lifecycle Governance and Updating Transparency

Given adaptive regulatory frameworks, explainability must remain stable and auditable across model revisions. Developers should incorporate version-controlled explanation logs, traceable concept evolution, and update-aware validation protocols. Predetermined update pathways should specify how interpretability constraints are preserved during retraining. Transparent lifecycle documentation is critical for regulatory compliance, post market surveillance, and medico-legal accountability.

#### 6.4.5. Strengthening Ethical Robustness and Equity Safeguards

Bias detection and mitigation should be embedded within explanation workflows rather than treated as external audits. Explainability tools can function as bias diagnostics by revealing spurious feature reliance or demographic sensitivity. Future datasets must report comprehensive demographic metadata to enable subgroup-level validation. Additionally, safeguards against automation bias including uncertainty visualization and decision deferral mechanisms should be incorporated to preserve clinician oversight and prevent overreliance.

Collectively, these strategic directions reposition explainability from an auxiliary feature to a foundational component of dependable clinical AI infrastructure. Progress will depend on unifying semantic alignment, calibrated uncertainty, prospective validation, and lifecycle governance within cohesive translational frameworks. Only through such integration can an explainable AI transition from promising experimental systems to reliable partners in oncological care.

## 7. Conclusions

While deep learning has redefined the technical landscape, most current evidence remains at Tier 1 or Tier 2 maturity. Transitioning to a routine clinical partner requires moving beyond ‘superficial transparency’ toward Tier 3 prospective evidence, where epistemic alignment and uncertainty awareness are validated in live oncology environments. The next phase of research must move beyond visualization-centric paradigms toward semantically grounded, prospectively validated, and regulation-ready systems. Artificial intelligence will not become a trusted clinical partner through performance alone; it will do so only when its reasoning is intelligible, its uncertainty is explicit, and its integration respects the epistemic and ethical foundations of oncology practice. Ultimately, bridging the trust gap is not a technical refinement; it is a structural redefinition of how intelligent systems are conceived, evaluated, and governed in medicine.

## Figures and Tables

**Table 1:** Landscape of deep learning applications in cancer imaging.

Ref.	Imaging Modality	Cancer Site	Clinical Task	Model Architecture	Performance Metrics	Challenges and Limitations	Dataset Used	Dataset Category
Clinical Decision Support (CDSS) and Interpretation								
[55]	Ultrasound	Breast	Clinical decision support	CNN + U-Net	Accuracy: 81%	Automation bias: Risk of overreliance; Cognitive load: Visual complexity of localization	Breast ultrasound (780 images)	Public
[56]	Mammography	Breast	Enhance diagnosis and interpretation	VGG/Inception/ResNet	Test Accuracy: 76%	Method instability: LIME varies across runs; Inconsistency: Lower accuracy of explanations	CBIS-DDSM	Public
[57]	Whole Slide Imaging	Kidney	Concept learning/Survival analysis	GNNs	AUC: 0.789; C-Index: 0.725	Concept overlap: Difficulty defining distinct concepts; Rarity: Rare concepts show lower performance	TCGA-RCC	Public
[58]	Ultrasound, MRI	Prostate	Early-stage detection; explanation of decision rationale	Pretrained DL (VGG-16, ResNet, Xception, etc.) + shallow ML (SVM, RF, etc.)	US Acc: 99%; MRI Acc: 87.5%	Local surrogacy: Requirement for local data; Input quality: Dependence on image quality	Prostate-MRI-US-Biopsy	Public
Image Recognition and Object Detection								
[59]	Biomedical images	Bladder	Automatic image recognition, grade/contour recognition	Deep Belief Network (DBN), ConvNeXt, RegNet, MaxViT	Accuracy: 98.75%; F1: 98.57%	Interpretability thresholds: Gaps in existing models; Constraints: Real-world noise applicability	Bladder cancer classification	Public
[39]	MRI	Brain	Automated object detection	YOLOv11 with Attention	mAP50: 96.8%	Intrinsic Limitation: Study lacks dedicated XAI (LIME/SHAP); Hardware: Constraints on inference	Brain Tumor (Kaggle)	Public
[60]	Histopathology	Breast	Detection through feature extraction	Modified DenseNet169	Accuracy: 99.50%; F1: 99.42%	Interpretability gap: Absence of advanced features; Case sensitivity: Difficulty with subtle benign cases	BreakHis, BACH, BHI	Public
[61]	Histopathology	Lung	Cancer detection	EfficientNetB3, Custom CNN	Accuracy: 95.1%; F1: 89.5%	Coarseness: Grad-CAM explanations can be non-specific; Sensitivity: To non-malignant features.	TCGA and LC25000	Public
[62]	Whole Slide Imaging	Lymph node	Tumour tissue detection	Custom CNN, VGG19	Accuracy: 0.9683	Unreliability: Methods sensitive to superpixel parameters; Complexity: High computational intensity	PatchCamelyon dataset (P-CAM)	Public
[63]	mpMRI	Prostate	Lesion detection and characterization	Cascaded FCN	sensitivity: 93.9%	Redundancy: Inefficiency for obvious cases; Bias: Reference standard bias in multi-institutional studies	11 MR devices; 3 institutions	Multi-institutional clinical data
[64]	Abdominal CT	Vertebra	Metastasis classification	EMCD + DenseNet201	Accuracy: 85.79%; AUC: 0.93	Alignment: Uncertainty maps may lack clinical alignment; Calibration: MCDO/DE limitations	Severance Hospital cohort	Private/Internal
Screening, Early Detection and Mortality Prediction								
[65]	Mammography	Breast	Organized screening	Deep CNNs (Vara MG)	BCDR: 6.7/1000; PPV: 17.9%	Lack of software interoperability, Binary confidence constraint, Initial feature gaps	Description of the 463,094 women	Multi-institutional clinical data
[66]	SERS Biosensing (SEARCH Chip)	Liver	Early detection and staging	Self-Learning CNN	AUC: 0.97; Accuracy: 0.87	Thresholds: Dimensionality reduction below 20 features reduces accuracy; Actionability gap	Serum samples (300 subjects)	Multi-population cohort consisting of serum samples from 300 subjects
[67]	CT	Lung	Early detection and CAD	LCxNet (Custom CNN)	Accuracy: 99.39%; F1: 99.40%	Ambiguity: Grad-CAM heatmaps can be diffuse; Validation: Needs multi-metric expert review	IQ-OTH/NCCD	IQ-OTH/NCCD lung cancer dataset
Segmentation and Analysis								
[68]	Multimodal MRI	Brain	Segmentation and analysis	CausalX-Net (DL + SCM)	DSC: 93.2%; HD95: 0.91 mm	Assumptions: High dependency on causal assumptions; Complexity: Implementation difficulty	BraTS 2021; ISLES 2017	Multi-institutional clinical data
[69]	MRI	Brain	Segmentation	3D U-Net	Dice Coefficient: 0.73	Constraint Gap: Lack of spatial constraints; Bias: Reliance on signal intensity	Institutional brain tumour data	Private/Internal
[70]	Ultrasound	Breast	Segmentation and classification	HyFormer-Net	Accuracy: 93.2%; Dice: 0.902	Baseline Diffuseness: Grad-CAM limitations; Validation: Lack of quantitative validation	BUSI; BUS-UCLM	Public
[42]	Ultrasound	Breast	Image segmentation	LIME/SHAP	Accuracy: 72.0%	Inconsistent feature attribution across models	Breast ultrasound images	Public
[71]	CT, Chest X-ray	Lung	Classification and segmentation	Proto-Caps	Accuracy: 95.3%	Persuasiveness Risk: Users may be misled if model is wrong; Complexity: High memory demands	LIDC-IDRI, CheXpert	Public
Tumor Classification and Grading								
[72]	MRI	Brain	Multiclass classification	SSPANet	Accuracy: 97%; Kappa: 95%	Coarseness: Grad-CAM coarse focus; Disconnect: XAI often disconnected from core architecture	Figshare Brain Tumor	Public
[73]	MRI	Brain	Glioma grading and localization	ResNet-50, 3D DeepSeg	Accuracy: 98.62%; Dice: 92	Voxel Gaps: Grad-CAM low-resolution; Noisy: Vanilla Gradient produces noisy visualisations	BraTS 2019/2021	Public/multi-institutional clinical data
[74]	3D mpMRI	Brain	3D brain tumour classification	MProtoNet	Bal. Accuracy: 0.870	Localization Coherence: Poor Grad-CAM performance; Complexity: 3D localization issues	BraTS 2020	Public
[40]	MRI	Brain	Detection and Classification	YOLOv11 (Two-stage)	Accuracy: 92.6%; F1: 0.899	Sensitivity: To MRI variations; Manual Dependency: Gap in automated bridging	BTDM, BTDS datasets	Public
[75]	FLAIR MRI, US	Brain, Breast	Tumour classification	SpikeNet (Hybrid)	Accuracy: 98.23%; AUC: 0.996	Visual Inaccuracy: Grad-CAM boundary spillover; Noise: Fragmented SHAP/LIME maps	TCGA–LGG; BUSI	Public/multi-institutional clinical data
[3]	Ultrasound	Breast	Classification	Hybrid Model Fusion	Accuracy: 97.14%; F1: 97.18%	Visual Constraint: Lacks automated reasoning support; Reasoning gap: Traditional XAI limits	Ultrasound breast images	Public
[76]	Ultrasound	Breast	Classifying tumours; associations with clinical descriptors	Multitask DL (VGG/ResNet encoders)	Accuracy 88.9%, Sensitivity 83.8%, and Specificity 92.3%	Clinical Trustworthiness Inter-observer Variability Alignment with Medical Practice Information Insufficiency	BUSIS dataset	Public
[36]	H&E Stained Images	Breast	Subtyping and classification	HACT-Net	Weighted F1: 84.15%; Weighted Accuracy: 63.21%; Concordance: 90%	Sensitive to entity detection accuracy	BRACS; BACH	Public/multi-institutional
[77]	Tomosynthesis (DBT)	Breast	Shape-based classification of lesions	8 Pretrained CNNs	AUC: 98.2%	Coarseness: Grad-CAM coarseness; Unclear: LIME visually less clear	39 breast DBT exams	Private/Internal
[78]	Mammography	Breast	Binary classification	CNN	Accuracy: 0.9675; AUC: 0.9937	Partial Explanation: Lack of proof vs. confidence; Opacity: “Black Box” problem	RSNA-Breast-Cancer	Public
[79]	Histopathology	Breast, Colon	Tumour classification	Simple/Mini-GoogLeNet	Accuracy: (High)	Calibration: Uncertainty miscalibration; Latent space: “Black Box” latent representation	CAMELYON17; AIDA-LNCO	Public/multi-institutional
[80]	Histopathology; Tomosynthesis; X-ray	Breast, Lung	Classification	ResNet (18/34/50)	Accuracy 97.61 Expected Calibration Error (ECE) 0.0095	Overconfidence: Confidence gap bias; Scale: Dataset scale sensitivity	BreakHis, BCS-DBT, Lung	Public/multi-institutional
[81]	Histopathological (Herlev) and Cytological (CIVa) images	Cervix, Ovary	Multiclass classification	RIRXEnsemble	Accuracy: 99.88%; AUC: 1.00	Perturbation: LIME reliance on random samples; Logic: Grad-CAM lacks clinical reasoning.	Herlev, CIVa, Mendeley	Public
[82]	Histopathology	Colon	Colon cancer diagnosis	Few-shot (ProtoNet)	Accuracy: 98.5%; AUC: 1.00	LIME Stability: Random perturbation reliance; Resolution: Grad-CAM coarse highlighting.	LC25000; EBHI	Public/multi-institutional
[37]	Tissue Images/WSIs	Multi-organ	Classification and grading	SCUBa-Net (GNN)	Accuracy: 93.0%; F1: 0.841	Latency: High computational complexity; Resolution: Explanation resolution differences	Colorectal, Prostate, Gastric	Public/multi-institutional
[83]	Whole Slide Imaging	Colorectal, Bone	Classification	AAOXAI-CD (Ensemble)	Accuracy: 9.42%; F-Score: 98.87%	Trade-off: Accuracy-interpretability trade-off; Dependency: Perturbation dependency	Warwick-QU; Osteosarcoma	Public
[84]	Histopathology	Lung	Classification	Bayesian Xception	Accuracy: 94.1%; AUC: 0.94–0.96	Threshold Sensitivity: OOD false confidence; Coverage: Reduced data coverage during abstention	TCGA, CPTAC, Mayo Clinic	Public/Private/Multi-inst.
[85]	CT	Lung	Identification and classification	FVCM-Net (Federated)	Accuracy: 97.66%; AUC: 0.995	Tuning Sensitivity: Manual tuning requirements; Bias: Static client weighting.	LIDC-IDRI, IQ-OTH/NCCD	Public
[20]	Biparametric MRI	Prostate	Grading aggressiveness	3D VGG/ResNet/ViT	AUC: 0.73	Context Gap: Lack of spatial context; Variability: Protocol/scanner variation.	ProstateNet dataset	Public
[86]	Dermoscopy	Skin	Lesion classification	DenseNet, ResNet	Accuracy: 93.28%; AUC: 99.64%	Semantic Gap: Pixel-based maps lack reasoning; Sensitivity: High hyperparameter dependency	HAM10000	Public
[87]	Dermoscopy	Skin	Detection and classification	VT-CNN	Accuracy: 99.89%; F1: 99.389%	Interference: Grad-CAM background noise; Compensation: Lack of HOA-XAI compensation	ISIC-2019, HAM10000	Public
[88]	Dermoscopy	Skin	Multiclass classification	XceSCNN	Accuracy: 92.643%	Approximation: Requirement for SHAP approximation; Subjectivity: Interpretation difficulty	ISIC	Public
[89]	Dermoscopy	Skin	Recognize lesion types	ResNet + ABELE	Bal. Accuracy: 0.838	Latency: Time-consuming extraction; Quality: Dependent on autoencoder reconstruction	ISIC 2019	Public
[90]	Dermoscopy	Skin	Categorization	ResNet-50/VGG16	Accuracy: 87–96%	Approximation: Local emphasis approximation issues; Robustness: Concerns in manual correction	ISIC and HAM10000	Public
[91]	Histopathology	Skin	Margin classification	ViT Transfer Learning	Accuracy: 0.928	Opacity: Intrinsic non-interpretable nature; Trust Gap: Subjective interpretation	Histopathological slides (50 pts)	Public

**Table 2:** System-level barriers across the deep learning development–deployment pipeline in oncological imaging.

Broad Obstacle Domain	Subcategory	Technical Constraints	Trade-Offs	Clinical Implication
Data availability, Quality and bias	Dataset scarcity and imbalance	Small sample sizes for rare cancers; minority underrepresentation; fragmented health records	Statistical power vs. generalisability	Inflated performance in development cohorts; failure in real-world deployment
	Annotation burden and subjectivity	Manual segmentation; pixelwise labelling; inter/intraobserver variability	Annotation fidelity vs. scalability	Limits dataset growth: inconsistent ground truth undermines trust
	Acquisition heterogeneity	Scanner/vendor bias; staining variability; resolution differences	Dataset diversity vs. distributional stability	Domain shift across institutions; degraded external validation
Model architecture and Computational complexity	Model scale and capacity	Large parameter counts; quadratic self-attention; long training times	Expressivity vs. feasibility	High-performing models impractical for clinical infrastructure
	Memory and efficiency constraints	Gigapixel WSIs; 3D context handling; client-side memory limits	Spatial resolution vs. deployability	Reduced resolution or patching compromises spatial reasoning
	Optimization stability	Vanishing gradients; unstable losses; manual hyperparameter tuning	Training stability vs. architectural flexibility	Reproducibility challenges across centres
Generalization and robustness	Distribution shift and OOD data	Non-IID clinical data; temporal drift; confounding variables	Robustness vs. dataset specificity	Performance decay after deployment
	Sensitivity to perturbations	Noise, artifacts, color statistics shifts	Sensitivity vs. feature richness	Erratic predictions in routine clinical settings
	Cross-site variability	Single-centre studies; single-vendor training	Controlled performance vs. external validity	Limited regulatory acceptance
Interpretability, Explainability and human factors	Black-box opacity	Deep feature abstraction; unclear decision logic	Predictive accuracy vs. interpretability	Clinician reluctance to adopt AI outputs
	Explanation instability	Saliency inconsistency; scattered attention maps	Transparency vs. reliability	Undermines confidence in safety-critical decisions
	Human subjectivity	Subjective visual validation; low interrater agreement	Human intuition vs. algorithmic rigor	Inconsistent evaluation standards
Clinical integration and workflow constraints	Workflow disruption	Manual pre/postprocessing; lack of automation	Model precision vs. usability	Poor adoption despite strong benchmarks
	Device and infrastructure limits	Hardware constraints; lack of clinical-grade devices	Model sophistication vs. bedside deployment	Restricts real-time or point-of-care use
	Contextual incompleteness	Image-only analysis; missing clinical metadata	Task simplicity vs. clinical realism	Reduced decision relevance
Regulatory, Ethical and Legal barriers	Privacy and governance	compliance; restricted data sharing	Privacy vs. reproducibility	Limits multi-institutional validation
	Accountability and liability	Medico-legal responsibility; risk of automation bias	Autonomy vs. oversight	Slows approval of autonomous systems
	Regulatory lag	High certification costs; evolving standards	Innovation speed vs. safety assurance	Delays translation into standard of care
Evaluation, validation and trustworthiness	Calibration and uncertainty	Overconfident predictions; epistemic uncertainty	Sensitivity vs. reliability	Unsafe decision-making in high-risk oncology
	Metric inadequacy	Lack of standard reliability metrics	Benchmark simplicity vs. clinical relevance	Misleading performance claims
	External validation gaps	Retrospective designs; selection bias	Study control vs. real-world validity	Weak evidence for deployment

**Table 3:** Comparative overview of XAI approaches in oncology imaging.

Category	Approach	Strengths	Limitations	Suitability in Oncology
Post hoc explainability	Gradient-based (Grad-CAM, Grad-CAM++)	Computationally efficient; architecture-agnostic; visually intuitive localization	Coarse spatial resolution; sensitive to input noise; may highlight non-pathological artifacts (e.g., surgical markers, scanner bias)	Screening and initial tumour localization (e.g., mammography, ultrasound) where rapid visual verification is required
	Perturbation-based (LIME, SHAP)	Strong local fidelity (LIME); theoretically grounded feature attribution (SHAP); supports multimodal inputs	Computationally expensive; sensitive to segmentation granularity; instability across repeated runs	Multimodal analysis combining imaging with genomic/clinical data to identify prognostic drivers
	Attention-based (Vision Transformers)	Captures global context; does not require pixel-level annotations; effective for large images	Limited interpretability of attention weights; unclear causal relationship between attention and prediction	Whole-slide histopathology analysis where tissue architecture is critical
Intrinsic/hybrid interpretable models	Concept Bottleneck Models (CBMs)	Clinically interpretable; supports human-in-the-loop refinement; aligns with medical ontologies	High annotation burden; risk of concept leakage; dependent on concept quality	Staging and prognosis tasks (e.g., TNM-based cancer assessment)
	Prototype-based (ProtoPNet, MProtoNet)	Provides intuitive “this looks like that” reasoning; links decision to exemplars	Limited flexibility with fixed prototypes; challenges in multimodal fusion	Diagnostic classification (MRI, mammography) aligned with radiologist reasoning patterns
	Disentangled representations	Improves robustness to domain shift; enables interpretable feature isolation	Latent factors may lack direct clinical meaning; interpretability not always guaranteed	Biomarker discovery and cross-site generalization (e.g., PET, MRI)

**Table 4:** Comparative overview of post hoc explainability methods in oncological deep learning.

Ref.	Clinical Objective	Deep Learning Architecture	Explainability Method	Reported Validation of Explainability	Performance Metrics	Reported Explainability Limitations	Dataset Used	Dataset Category
[3]	Early cancer detection	Hybrid Deep Fusion: VGG16 + DenseNet121 + Xception	Grad-CAM++	Visually consistent with expert assessment	Accuracy: 97.14%; F1: 97.18%	Reasoning Gap: Lacks automated clinical reasoning support; limited degree of interpretability beyond visual overlays	Ultrasound breast images	Public
[7]	Explainable diagnosis and trust	ResNet50 and DenseNet121 (Pretrained)	Grad-CAM	Localized core pathological regions	Accuracy: 94.3%; AUC: 0.99	Coarseness: Diffuse activations; Semantic inconsistency: High probability of highlighting non-salient features	Brain MRI; Chest X-ray	Public
[37]	Histopathology classification	SCUBa-Net (GCN + Transformer)	Grad-CAM	Activation maps aligned with histopathology	Accuracy: 93.0%; F1: 0.833	Computational overhead: Slow inference generation; Propagation bias: Sensitivity to graph node aggregation logic	Colorectal, Prostate, Gastric, Bladder	Public/multi-institutional
[36]	Breast tumour subtyping	HACT-Net (Hierarchical GNN)	GraphGradCAM	Feature attribution aligned with pathology	Weighted F1: 84.15%; Accuracy: 63.21%	Structural dependency: Explanation fidelity sensitive to the accuracy of the underlying entity detection nodes	BRACS; BACH	Public/multi-institutional
[59]	Efficient bladder screening	ConvNeXt + RegNet X + MaxViT + DBN	SHAP	Identified individual feature contributions	Accuracy: 98.75%; F1: 98.57%	Computational complexity: SHAP approximation requirements reduce global explanation fidelity	Bladder cancer classification	Public
[86]	XAI effectiveness in skin cancer	DenseNet, ResNet, MobileNet	Integrated Gradients, SHAP, LIME	Mathematical saliency regions vs. clinical meaningfulness	Accuracy: 93.28%; AUC: 99.64%	Semantic Gap: Pixel-based maps do not translate to semantic ABCDE criteria; Robustness: High hyperparameter sensitivity	HAM10000	Public
[56]	Trust enhancement via diagnostics	ResNet50 (fine-tuned)	Grad-CAM, LIME, SHAP	Hausdorff distance to expert ROIs	Test Accuracy: 76%	Method Instability: LIME is unstable across runs; Inconsistency: Lower accuracy of explanations compared to model	CBIS-DDSM (Mammography)	Public
[88]	Multiclass skin classification	XceSCNN (XCovNet + SCNN + ELANet)	SHAP	Global SHAP plots of feature influence	Accuracy: 92.643%; F1: 96.47%	Complexity: SHAP becomes inefficient for large models; Approximation: Requirement for approximation reduces fidelity	ISIC Dataset	Public
[81]	Early multiclass cell classification	RIRXEnsemble (ResNet + InceptionResNet + Xception)	Grad-CAM, LIME	Confidence Drop; 3D visualization (t-SNE)	Accuracy: 99.88%; AUC: 1.00	Perturbation Bias: LIME reliance on random samples; Logic Gap: Grad-CAM lacks complete clinical reasoning logic	Herlev, CIVa, Mendeley LBC	Public
[62]	Tumor detection explanation	Custom CNN + VGG19	LIME (SLIC, FHA, etc.)	Heatmaps aligned with expert knowledge	Accuracy: 0.9683	Unreliability: Super pixel methods are sensitive to parameters; Granularity: May lack global contextual reasoning	Patch Camelyon (P-CAM)	Public
[73]	Transparent brain diagnosis	ResNet-50 + 3D DeepSeg	NeuroXAI (VG, GBP, IG, GIG, SmoothGrad, Grad-CAM)	Network inspection of hierarchical detection	Accuracy: 98.62%; Dice: 92	Noisy visuals: Vanilla Gradient is noisy; Resolution: Grad-CAM suffers from low-resolution heatmaps	BraTS 2019 and 2021	Public/multi-institutional
[61]	Interpretable pathology support	Bespoke CNN + EfficientNetB3	Grad-CAM	Alignment between focus and annotations (0.78)	Accuracy: 95.1%; F1: 89.5%	Spatial resolution gap: Diffuse heatmaps lacking pathological specificity	TCGA and LC25000	Public
[60]	Feature-driven cancer detection	Modified DenseNet	CAM, Saliency Map	Comprehensive reasoning via CAM/Saliency	Accuracy: 99.50%; F1: 99.42%	Resolution constraint: Inability to delineate fine morphological features required for subtle case differentiation	BreakHis, BACH, BHI	Public
[83]	Explainable cancer classification	Faster SqueezeNet + RNN Ensemble	LIME	Clear explanations for black-box predictions	Accuracy: 99.42%; F1: 98.87%	Perturbation dependency: Results depend on sampling; Trade-off: Accuracy-interpretability trade-off	Warwick-QU, Osteosarcoma	Public
[72]	Context-aware detection	SSPANet	Grad-CAM, Grad-CAM++, EigenGradCAM	Noise-free heatmaps aligned with anatomy	Accuracy: 97%; Kappa: 95%	Architectural disconnect: Disparity between XAI output and core feature design; Low-rank approximation limits	Figshare Brain Tumor	Public
[85]	Privacy-preserving detection	FVCM-Net (VGG16 + CBAM)	SHAP, HiRes-CAM	Boundaries confirmed by radiologist	Accuracy: 97.66%; AUC: 0.995	Manual parametrization: High sensitivity to kernel width and sampling hyperparameters	LIDC-IDRI, IQ-OTH/NCCD	Public
[50]	Personalized survival risk	CVAE-based DySurv with LSTM	Permutation importance	Time-dependent concordance metrics	C-Index: 70.4%; IBS: 0.122	Interaction blindness: Failure to account for non-linear feature interactions in risk attribution	MIMIC-IV and eICU	Public
[66]	Monitoring of early-stage hepatocellular carcinoma	Self-Learning CNN	SHAP (Feature extraction)	Reduced dimensionality by 95%	AUC: 0.97; Accuracy: 0.87	Thresholds: Accuracy drops if reduction is too aggressive; Actionability: Gap between SHAP values and clinical action	Serum samples (300 subjects)	Multi-institutional

**Table 5:** Comparative summary of non-post hoc explainable deep learning methods in oncology.

Ref.	Clinical Objective	Deep Learning Architecture	Explainability Method	Reported Validation of Explainability	Performance Metrics	Reported Explainability Limitations	Dataset Used	Dataset Category
[76]	Clinically aligned CAD systems	Multitask learning backbone	BI-RADS descriptors + tumour class	Descriptors consistent with clinical practice	Accuracy: 88.9%;	Lexicon restriction: Explanations constrained by a predefined medical vocabulary	BUSIS; BUSI	Public
[89]	Exemplar-based trust enhancement	ResNet + ABELE (PGAAE)	ABELE (Exemplars and Saliency)	+22% confidence increase after correcting errors	Balanced Accuracy: 0.838; RMSE: 0.08–0.24	Latency: Explanation extraction is time-consuming; Quality: Highly dependent on autoencoder reconstruction fidelity	ISIC 2019	Public
[10]	High-performance explainable diagnosis	Concept Complement Bottleneck (CCBM)	Ante-hoc concept-based model	Faithfulness via concept intervention	AUC: 93.96%; ACC: 88.15%	Concept divergence: Discovered concepts may lack clinical semantic alignment; Potential concept leakage	Derm7pt, Skincon, BrEaST, LIDC-IDRI	Public/External
[82]	Explainable few-shot cancer diagnosis	ProtoNet (ConvNeXt-Tiny)	Grad-CAM, LIME, prototypes	Validated by panel of 4 medical professionals	Accuracy: 98.5%; ROC-AUC: 1.000	LIME Instability: Reliance on random perturbations; Grad-CAM Resolution: Lack of complete textual reasoning	LC25000; EBHI	Public/multi-institutional
[68]	Causality-aware tumour segmentation	CausalX-Net (3D U-Net + SCM)	Structural Causal Models	Counterfactual maps identified 81% of edema errors	Dice (WT): 93.2%; HD95: 0.91 mm	Complexity: High implementation difficulty; Assumptions: High dependency on causal assumptions	BraTS 2021; ISLES 2017	Public/multi-institutional
[71]	lung nodule classification	Proto-Caps (Capsule + Prototype)	Visual prototypes + attribute scores	Alignment confirmed via expert rater study	Accuracy: 95.3%; Faithfulness: 0.62	Persuasiveness Risk: Users may be misled if model is wrong; Complexity: High memory/computational demands	LIDC-IDRI; CheXpert	Public
[74]	3D case-based tumour classification	MProtoNet (3D ResNet + online-CAM)	Case-based reasoning (prototypes)	Improved localization over Grad-CAM	Balanced Accuracy: 0.870 ± 0.021	Fixed Assignments: Prototype assignments are fixed; Fusion Gaps: Difficulty analyzing individual modalities	BraTS 2020	Public
[57]	RCC subtyping and survival prediction	GNN-based Concept Learning	Concept Bottleneck Models (CBMs)	High-risk concepts linked to mortality	Balanced Acc: 0.682; C-Index: 0.725	Concept overlap: Difficulty defining distinct local concepts; Rarity: Rare concepts show lower performance	TCGA-RCC	Public
[156]	Bias-aware interpretable profiling	MorphoGenie (VAE + GAN)	Disentangled learning	Outperformed baseline VAEs in reconstruction	AUC (Actin): 0.87; Nucleoli: 0.83	Interpretation Gap: Latent features lack clear morphological interpretation; Reductionist Framework	LC, CPA, CCy, EMT	Public/Private
[87]	Automated skin cancer detection	OXAI-SCC-Net	Grad-CAM guided NAS; HOA-XAI	Highlighted medically relevant borders	Accuracy: 99.89%; F1: 99.389%	Interference: Grad-CAM includes background noise; Lack of compensation: Missing HOA-XAI compensation	ISIC-2019; HAM10000	Public
[70]	Joint lesion segmentation/classification	HyFormer-Net	Intrinsic Attention + Grad-CAM	Mean IoU 0.86 (attention-boundary alignment)	Accuracy: 93.2 ± 0.6%; Dice: 0.902	Baseline diffuseness: Coarse localization of Grad-CAM; Attention over-smoothing	BUSI; BUS-UCLM	Public
[73]	Voxelwise brain tumour segmentation	3D U-Net + Multinomial Dirichlet	NeuroXAI (VG, GBP, IG, Grad-CAM)	Uncertainty maps identified model ignorance	Accuracy: 98.62%; Dice: 92	Noisy visuals: Vanilla Gradient noise; Resolution: Grad-CAM suffers from low-resolution heatmaps	BraTS 2019/2021	Public/multi-institutional
[64]	Vertebral metastasis detection	EMCD (YOLOv5m + DenseNet201)	Uncertainty-CAM	Accuracy improved to 95.68% (50% retention)	Accuracy: 85.79%; AUC: 0.93	Clinical alignment: Uncertainty maps may not perfectly align clinically; MCDO/DE limitations	Severance Hospital cohort	Private/Internal
[84]	Confidence-calibrated prediction	DCNN (Xception/ResNet) + MCDO	Uncertainty thresholding	Experts confirmed biological relevance of maps	AUROC: 0.945–0.966; Acc: 94.1%	Reduced coverage: ~25% cases abstain; Sensitivity: High threshold sensitivity and cost	TCGA, CPTAC, Mayo	Public/Private/multi-institutional
[67]	Deployable early cancer detection	LCxNet (Custom CNN)	Grad-CAM, t-SNE	t-SNE proved linear separability of features	Accuracy: 99.39%; F1: 99.40%	Spatial ambiguity: Low-resolution Grad-CAM heatmaps; Lack of structured evidence	IQ-OTH/NCCD; LC25000	Public
[77]	Morphological shape classification	Pretrained CNN Ensemble	Grad-CAM, LIME, t-SNE	Correct identification improved performance	AUC: 98.2%	Coarseness: Grad-CAM produces coarse maps; Visual clarity: LIME super pixels are visually less clear	39 breast DBT exams	Private/Internal

**Table 6:** Clinical explanation alignment and misalignment across domains.

Domain	Clinically Meaningful Explanation (Requirement)	Decision Pathway (Alignment)	Primary Failure Mode (Misalignment)
Breast ultrasound	Localization of microcalcifications and spiculated margins	Alignment with BI-RADS descriptors for benign/malignant classification	High sensitivity to visual noise and artifacts, leading to false positives
Prostate MRI	Highlighting diffusion restriction and T2-weighted signal changes	Integration of PI-RADS v2.1 logic and contextual data (PSA, patient history)	Scanner/vendor variability and inability to resolve equivocal PI-RADS 3 lesions without context
Digital pathology	Translation of pixels into cellular/glandular constructs	Multiscale reasoning (5× to 20×) from cellular morphology to tissue architecture	Shortcut learning from staining protocols and annotation sparsity in gigapixel slides
Multimodal oncology	Correlation between metabolic uptake (PET) and anatomical detail (CT)	Alignment of macroscale patterns with genomic/longitudinal trajectories	Overfitting on small, paired datasets and missing temporal data points

**Table 7:** Distinguishing “visually persuasive” from “trust calibrating” explanations.

Attribute	Visually Persuasive Explanations	Trust Calibrating Explanations
Common methods	Grad-CAM, Saliency maps	Concept bottleneck models, Uncertainty maps
Primary goal	Highlighting where the model looks	Clarifying why the model is certain or uncertain
Cognitive impact	Increases load through visual clutter and “authority modulation”	Streamlines triage by flagging high-entropy cases for review
Trust outcome	Risk of “illusion of interpretability” and automation bias	Promotes epistemic alignment with clinical reasoning
Success metric	Plausibility (looks right to the eye)	Faithfulness/Fidelity (reflects true model logic)

## Data Availability

Data sharing is not applicable to this article as no new data were created or analyzed in this study.
